# 7 tesla multiscale entropy analysis reveals increased resting-state complexity in key regions for fear and anxiety in spider-fearful individuals

**DOI:** 10.1016/j.neuroimage.2025.121371

**Published:** 2025-07-13

**Authors:** Matthias Grieder, Niklaus Denier, Kay Jann, Werner Strik, Leila M. Soravia, Kristina Adorjan, Marcel Meyer, Elisabeth Jehli

**Affiliations:** a Translational Research Center, University Hospital of Psychiatry and Psychotherapy, University of Bern, Bern, Switzerland; b Laboratory of FMRI Technology, USC Stevens Neuroimaging and Informatics Institute, Keck School of Medicine, University of Southern California, Los Angeles, CA, USA; c University Hospital of Zurich, Department of Neurosurgery, Zurich, Switzerland

**Keywords:** Multiscale entropy, Complexity, Resting state, FMRI, Spider phobia, 7 Tesla

## Abstract

Resting-state functional connectivity is limited in assessing the temporal dynamics of brain networks and, due to an insufficient signal-to-noise ratio, in detecting subtle changes at 3T MRI. Nonetheless, measures of complexity, which capitalize on temporal dynamics, have revealed alterations for some affective disorders at this field strength. Anxiety disorders have received only scant attention in this regard, despite indications of altered functional brain architecture in spider-fearful participants (SP). To address this gap, we probed resting-state complexity using 7T MRI, comparing 28 adults with SP with 45 healthy controls (HC). We computed multi-scale entropy (MSE) on ten scales (1 – 0.1 Hz) for brain regions of the fear and anxiety networks in HC and SP. The MSE scales interacted with group (HC, SP) and brain area, revealing MSE increments in limbic regions in SP (versus HC). Whilst most MSE changes related to SP ranged between 1 and 0.33 Hz, the MSE of the bed nucleus of the stria terminalis (BNST), a nucleus involved in anxiety and the hormonal system, exhibited increases on all scales bar two for SP (i.e., for 0.5 – 0.125, and 0.1 Hz). MSE was also positively associated with SP severity (but not trait anxiety) in the BNST. Altogether, 7T fMRI detected elevated MSE in SP, indicating excessive intra-regional processing in brain regions key to fear and anxiety. The most pronounced effects were found in the BNST, corroborating its central role in the anxiety circuit.

## 1. Introduction

The human resting state arises from an intrinsic brain activation architecture, ofttimes measured with functional magnetic resonance imaging (fMRI). Resting-state networks (RSNs) denote recurring mental states, which, in turn, emerge from synchronized activity – the correlation of neural activation fluctuations between segregated brain regions over time (Biswal et al., 1995; Raichle et al., 2001). RSNs have been linked to multiple primary cognitive functions and mirror overlapping patterns of functional connectivity corresponding to task-related activation topography (e.g., attention, Damoiseaux et al., 2006; Jann et al., 2010). The introduction of functional connectivity (FC) studies has generated substantial insights into diseased brain networks and consolidated markers for cognitive symptomatology. However, the FC measure yields only one correlative metric for a given scan, thereby failing to characterize the underlying temporal dynamics of complex brain systems. This major shortcoming might account for inconsistent findings across studies (Calhoun and Adali, 2012).

Resting-state fMRI complexity may help resolve such discrepancies. An apt metric of the complexity of fMRI signals is sample entropy – a statistical measure quantifying the complexity or irregularity of a timeseries signal. This method samples a limited number of data points with a predefined pattern length (*m*) and threshold (*r*) (Smith et al., 2014; Wang et al., 2014). Sample entropy reflects the rate of information generation or processing in complex systems. It aids in differentiating complex signals from random noise, thus improving the understanding of the dynamics in physiological systems and their links to pathology (Pincus, 1991; Yang et al., 2018). In the context of functional brain networks, entropy could indicate the transition or exploration between different states of segregated brain networks, resulting in a greater propensity for information processing (McDonough and Nashiro, 2014; Wang et al., 2018). When sample entropy is applied to a set of coarse-grained time series by averaging the original time series over a range of time scales, this is termed multiscale entropy (MSE). MSE detects the self-similarity of complex signals across multiple time scales in a random noise environment (Costa et al., 2002). Many complex systems in nature follow a 1/*f* power law (frequency [*f*]), with scale-invariant features (He et al., 2010). This also applies to the brain, with data from EEG, intracranial recordings (Medel et al., 2023) and fMRI mapping onto the 1/*f* power law (He, 2011). The main advantage of MSE is that it can assess alterations and interactions of neural circuits on spatial and temporal scales. Increasing temporal scales in the context of MSE reflect signal fluctuations at different frequencies, i.e. with increasing scale, the frequency declines with 1/(scale * TR). Accordingly, in contrast to sample entropy, the multiscale dimension of MSE allows capturing the degrees of complexity across different frequencies, in which segregated brain regions communicate locally or distally. For instance, previous work has suggested that higher frequencies mirror aspects of intra-regional information processing, whereas lower frequencies are supposedly linked to inter-regional information transfer (McDonough and Nashiro, 2014; Vakorin et al., 2011; Wang et al., 2018). MSE analyses of resting-state fMRI signals have recently begun to burgeon in basic and clinical neuroscience, showcasing that brain entropy is a measure of adaptability of a complex system and higher-order cognition, or, owing to its capability in capturing spatiotemporal dynamics, a candidate entity for a “common currency” that integrates neural and mental levels of the brain and the self (Bassett et al., 2006; Northoff et al., 2020; Omidvarnia et al., 2021).

This research illustrates that MSE may add profound insights based on the temporal dynamics over and above the FC components. In some studies, FC and MSE show converging results (McDonough and Nashiro 2014); yet, our work indicates that MSE might be more sensitive than FC (Grieder et al., 2018). Such discrepancies may be explained by the dependence of both FC and MSE on adequate temporal and spatial signal-to-noise ratios (SNR). With advances in processing algorithms, and especially the availability of ultra-high field 7T MRI, images with increased SNR permit detecting more subtle effects (Dumoulin et al., 2018; Pohmann et al., 2016). The relative gain in sensitivity and spatial specificity stems from increased tissue-specific functional responses. These responses are related to a shorter venous T_2_* (signal decay time constant), leading to higher extravascular yet lower intravascular signals – compared to 3T imaging (Uludağ et al., 2009). MSE capitalizes on these strengths but has not been explicitly probed with resting-state fMRI at 7T.

Addressing this gap, we extracted MSE using 7T MRI. To this end, fMRI data from a sample of spider-fearful participants (SP) and healthy controls (HC) was re-utilized (Jehli et al., 2024). This study found both increased and decreased FC related to SP, albeit less so at the group level. Following on from this, we examined whether MSE at 7T is sufficiently sensitive in detecting group differences without task-induced spider cues – characteristic of processing biases in SP.

Complexity analyses are still sparse for psychiatric disorders in the neuroimaging domain. Nonetheless, they hold promise for clinical cohorts – e.g., depression, attention deficit hyperactivity disorder, and schizophrenia – yielding effects on symptom severity or improvement (Denier et al., 2024; Fernández et al., 2013; Zhang et al., 2024). Thus far, the field of complexity has largely been neglected in anxiety disorders (Takahashi, 2013). Acute threat elicits fear instantaneously, guiding an adaptive response to escape, avoid, sit out, or confront the endangering source. Healthy fear response declines once the threat has disappeared; however, in individuals with a specific phobia, the fear response is maladaptive and characterized by an exaggerated, persistent fear of a phobic object or situation (LeBeau et al., 2010). Fear memories associated with phobic stimuli are also retrieved excessively, notably leading to sustained anxiety, marked by threat anticipation and distress (de Quervain and Margraf, 2008).

One common form of specific phobia is spider phobia, typified by an inadequate fear response towards spiders – the phobic object when encountered (Schweckendiek et al., 2011). In such situations, individuals with spider phobia show a hyperactive cerebral fear network, involving regions responsible for fear response and processing (Leehr et al., 2024). Spider phobia is related to hyperactivation in the amygdala, anterior cingulate cortex (ACC), insula, and thalamus and to reduced fear-controlling activation of the prefrontal cortex during confrontation with spider stimuli (Leehr et al., 2024). Predictably, most studies investigating functional brain activity of spider phobia applied a task-based approach, involving confrontation with phobic cues. By contrast, resting-state data on the functional architecture of spider phobia, i.e., data without cue-confrontation, is sparse (Leehr et al., 2024). Notably, spider phobia is an enduring condition, and evidence of the sustained anxiety of individuals with spider phobia (i.e., constant threat anticipation) emphasizes the importance of investigating alterations in both the fear and anxiety networks at rest (Münsterkötter et al., 2015). In addition to the fear response, individuals with spider phobia experience phobic anxiety, that is, a persisting anxious anticipation of a potential threat (Münsterkötter et al., 2015). Given that both fear and anxiety RSNs appear to be altered in spider phobia, it is imperative that this study examines the brain circuits involved in phasic fear as well as sustained anxiety (Davis et al., 2010; Jehli et al., 2024).

Prior findings of FC or brain activation related to spider phobia are only partially informative for work on resting-state complexity. This is because resting-state complexity measures signal self-similarity and information production rates. These are entities of system complexity and, thus, constitute a different modality from FC or brain activation. In view of the paucity of research on complexity in SP and the probable neural involvement of the fear and anxiety circuits, we focused on assessing region-specific MSE alterations in SP (Lebow and Chen, 2016). Based on a functional view of SP, several fear- and anxiety-related brain regions are likely altered compared to HC. As a control measure, we compared the mean MSE of whole-brain grey matter, where group differences were not expected.

Our regions of interest (ROIs) were defined based on research demonstrating their involvement in fear and anxiety networks. The fear response and processing network involves the amygdala, ACC, and insula (Schweckendiek et al., 2011). The fusiform gyrus, parahippocampal gyrus, and thalamus support object recognition and have been shown to interact with the amygdala, making them relevant for phobic fear (Nakataki et al., 2017; Soravia et al., 2016). Owing to the crucial role of fear memory in the apprehensive state of individuals with SP (de Quervain and Margraf, 2008), several studies have included the hippocampus in the fear network (for an overview, see Duval et al., 2015). Moreover, defensive and fear-controlling processes have been linked with the medial prefrontal (MPFC) and orbitofrontal cortices (OFC, Leehr et al., 2024; Schweckendiek et al., 2011). Collectively, the fear network comprises regions of the limbic and limbic-related system (amygdala, hippocampus, parahippocampal gyrus, thalamus, insula) along with the prefrontal (MPFC, OFC, ACC) and visual association cortices (fusiform gyrus).

Conversely, the anxiety circuit, according to Avery et al. (2016), includes areas such as the hypothalamus, the bed nucleus of the stria terminalis (BNST), the hippocampus, the amygdala, and the insula. Their meta-analysis revealed that the caudate nucleus might play a role in threat anticipation. Hence, the caudate nucleus, as part of the dorsal striatum, also merits inclusion in the current complexity analysis, not, however, the ventral striatum, which has typically been linked to social anxiety rather than spider phobia (Boehme et al., 2013). Altogether then, the anxiety circuit also includes regions of the limbic and limbic-related system (hypothalamus, the BNST, the hippocampus, the amygdala, and the insula) along with regions of the basal ganglia system (caudate nucleus).

In neuroimaging fMRI methodology, subcortical brain structures (e. g., thalamus, BNST, caudate nucleus) are inherently difficult to image, even at 3T, due to their small size and their location proximate to the liquor-filled ventricles. While 7T theoretically improves tSNR for cortical and subcortical areas, there are known effects of increased susceptibility at higher field strengths, specifically in subcortical structures with higher iron contents, which causes faster T2* decay. Additionally, subthalamic areas are located farther from the receive elements of standard head coils and thus underlie g-factor penalty as the sensitivity of individual coils is relatively homogeneous in the center of the brain. Thus, acceleration techniques such as GRAPPA and SENSE, exploiting different receive sensitivities, are not providing the same benefit as for cortical areas. Nevertheless, the increased spatial resolution and potential gain of tSNR at 7T presumably prove advantageous. Corroborating evidence stems from direct comparisons of 3T versus 7T: it has repeatedly been demonstrated that 7T specifically improves the tSNR for subcortical areas, thus supporting enhanced reliability of MSE computation over 3T in those areas (Colizoli et al., 2020; Lucas et al., 2023).

Recent data further attests to the advantages of 7T for imaging cortical and subcortical regions. Using ultra-high field imaging, we revealed that brain regions of the anxiety circuit, particularly those partly overlapping with the fear network, could also be altered in the functional architecture of SP and, possibly, its complexity (Jehli et al., 2024) – a finding not uncovered in the 3T literature so far. Note that the fear and anxiety circuits have not only been linked to specific phobias but also, at 3T, to depression, generalized and psychotic anxiety, and paranoia in schizophrenia (Buff et al., 2017; Goltermann et al., 2023; Strik et al., 2017). Research elucidating the complexity of SP is wanting, however, particularly at ultra-high field imaging.

This study, therefore, set out to, for the first time, investigate resting-state complexity in SP. Given the novelty of this work, the analysis must be considered exploratory rather than confirmatory. Regional MSE was used as the complexity metric, including mean MSE within each ROI of the fear and anxiety circuit as described above. We were interested in the interaction of the factor group (SP and HC) and the MSE of a set of frequency scales as a function of ROI (for details, see Section 2.4). To assess convergent validity, we expected a relationship between MSE and the severity of fear of spiders. Conversely, to verify discriminant validity, we correlated MSE with trait anxiety – here, we did not expect any significant associations. Taken together, this study aimed to introduce resting-state complexity measures to 7T fMRI as well as to anxiety disorders.

## 2. Methods

### 2.1. Study participants and procedures

The dataset of this study was drawn from Jehli et al. (2024). Ninety-four adults were screened for eligibility in this observational and open crossover study. Recruitment was accomplished via local newspapers (print and online) and social media advertisements. There were two versions of advertisements for recruitment that reached out either to individuals perceiving themselves as spider-phobic or to healthy adults. For instance, the headline of the advertisement for individuals with SP was *«Does the idea of seeing a spider or looking at a picture of a spider trigger panic in you (sweating, shortness of breath, trembling, racing heart, crying, etc.)?»* (translated from German). The attention of the healthy control group was drawn to the perceptual aspects of the study more broadly («Magnetic *resonance imaging study about perception»*.). In an initial phone call, potential participants were assigned to either the SP or HC group according to their own perception of being spider-phobic. To definitively subdivide the participants into groups with SP and without SP, the Fear of Spider Questionnaire (FSQ; Szymanski and O’Donohue, 1995) was performed during the testing session to assess SP severity. Inclusion criteria for the study were Swiss German or German mother tongue and an age range between 18 and 50 years. Individuals with a history of neurological or any other psychiatric disorders, current psychoactive drug use or drug abuse, sensory impairments, magnetic objects in or on the body (implants, pacemaker, etc.), claustrophobia, or pregnancy were excluded from the study – note: women underwent a pregnancy test prior to the MRI scan. In accordance with these criteria, 75 participants were included. Informed consent was obtained from all participants for being included in the study. This study received ethical approval from the cantonal ethics committee of Bern (KEK-Nr. 2020–01, 259). Those subjects meeting the inclusion criteria went on to complete a battery of questionnaires to conclude the screening. The Brief Symptom Checklist (BSCL; Franke, 2017) was used to assess psychological distress. General trait anxiety was measured using the State-Trait Anxiety Inventory (STAI; Spielberger, 1989). The study was also registered in the German registry of clinical trials (Deutsches Register Klinischer Studien, DRKS00023089).

### 2.2. MRI acquisition and preprocessing

MRI data was recorded using a 32-channel head/neck coil (Nova Medical, Wilmington, MA) in a Siemens Magnetom Terra 7T scanner (Siemens Healthcare, Erlangen, Germany) at the Translational Imaging Center, located at the perimeter of the University Hospital Bern in Switzerland. High-resolution T1-weighted anatomical images were collected with an MP2RAGE protocol using the following settings: number of sagittal slices = 256, voxel size = 0.63 × 0.63 × 0.63 mm, field of view = 240 × 240 mm, matrix size = 384 × 384, TR = 6000 ms, TE = 2.06 ms, TI_1_ = 800 ms, TI_2_ = 2700 ms, flip angle_1_ = 4°, flip angle_2_ = 5°, TA = 460 s. Resting-state fMRI scans were acquired with a multiband echo-planar protocol comprised of: number of measurements = 360, number of slices = 60, voxel size = 2 × 2 × 2 mm, field of view = 204 × 204 mm, matrix size = 102 × 102, TR = 1000 ms, and TE = 25 ms, TA = 360 s. Image preprocessing was performed using the CONN toolbox (Nieto-Castanon, 2021) and included motion realignment, slice-time correction, coregistration, normalization to MNI space, spatial smoothing with a Gaussian kernel (3 mm full-width half-maximum), high-pass detrending (0.006 Hz), and denoising (Behzadi et al., 2007). Mean framewise displacement (mean FD) was computed to compare the magnitude of change in head position over the resting-state scanning time (Power et al., 2012).

### 2.3. Subdivision of fear and anxiety brain regions into functional units

**Fig. 1.**
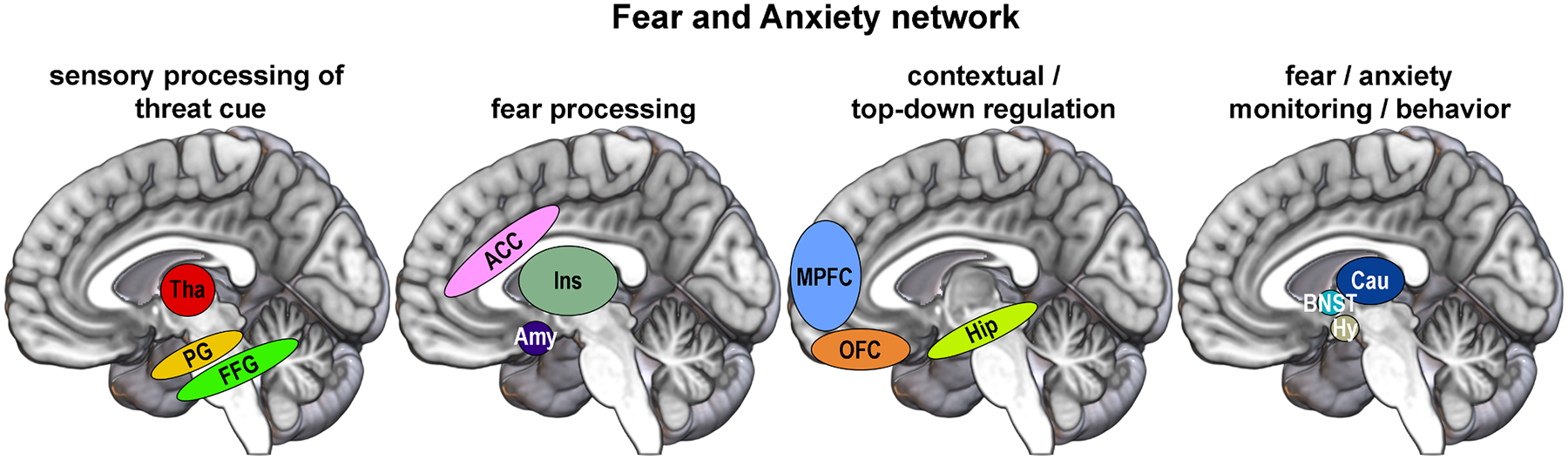
Fear and Anxiety network. Conjunct human fear and anxiety network brain regions, subdivided into functional units. ACC, anterior cingulate cortex; Amy, amygdala; BNST, bed nucleus of the stria terminalis; Cau, caudate nucleus; FFG, fusiform gyrus; Hip, hippocampus; Hy, hypothalamus; MPFC, medial prefrontal cortex; OFC, orbitofrontal cortex; PG, parahippocampal gyrus; Tha, thalamus.

As outlined in the introduction, the ROIs comprised brain regions of the fear and anxiety networks. In view of the substantial overlap of the fear and anxiety circuits, with either circuit appearing to play a key role in SP (see introduction), no strict segregation of the two networks was attempted in this study. Furthermore, this study did not assess the specific underlying functions of each ROI. Instead, using the literature as a guide, the convoluted set of ROIs was subdivided into functional units, rendering the findings more structured and meaningful relative to a single ROI pool. The statistics, however, were performed on the ROIs as one set. This is because such functional units add clarity and might be assembled differently. Fig. 1 illustrates these functional units of the fear and anxiety networks.

### 2.4. Complexity analysis

The preprocessed resting-state fMRI time series of each participant was subjected to the LOFT Complexity Toolbox (Jann, 2021) for voxel-wise MSE computation with a pattern matching threshold of *r* = 0.2, a pattern length of *m* = 2, and a time length scale of *a* = (1,2,3 … 10) with corresponding frequencies (1 Hz, 0.5 Hz, 0.33 Hz, 0.25 Hz, 0.2 Hz, 0.17 Hz, 0.14, 0.125 Hz, 0.111 Hz, 0.1 Hz) (Li et al., 2018; Smith et al., 2014; Sokunbi, 2014). All images were masked with a mean grey matter mask composed of all subjects. To extract individual ROI-specific mean MSE, 10 of the 12 ROI masks were generated using the automated anatomical labeling (AAL 1) in the WFU Pickatlas tool (v 3.0.5b, Maldjian et al., 2004, 2003; Tzourio-Mazoyer et al., 2002). As for the remaining two ROI masks, the BNST mask was adopted from Avery et al. (2014), and the hypothalamus mask, in turn, was adopted from the Talairach Daemon in the WFU Pickatlas tool (Lancaster et al., 2000). Altogether, the 12 ROIs consisted of bilateral masks of the ACC, amygdala, BNST, caudate nucleus, fusiform gyrus, hippocampus, hypothalamus, insula, MPFC, OFC, parahippocampal gyrus, and thalamus. The mean sample entropy value for each ROI, scale, and participant was extracted using a custom-written MATLAB script (R2022a, Mathworks, Natick, MA). To assess whether the MSE of SP and HC differed between the ROIs and the scales, a mixed 2 × 12 × 10 ANCOVA was conducted with group (HC/SP) as the between-subjects factor and ROI (12 ROIs) and scale (scales 1 – 10) as the within-subjects factors, respectively. Age and sex served as covariates, and Mauchly’s test for sphericity was used to indicate whether the resulting p-values needed Greenhouse-Geisser correction. Significant interactions were disentangled by performing independent *t*-tests investigating group differences of MSE within each ROI and scale. The MSE analyses were performed using the rstatix package (v 0.7.2) for the ANCOVA and the ggpubr package (v 0.6.0) for the *t*-tests in R (v 4.4.1). The post-hoc p-values were corrected for multiple testing using false discovery rate (FDR).

### 2.5. Relationship between complexity and fear

To assess whether complexity alterations are linked to trait anxiety or are specific to fear of spiders severity, a non-parametric Spearman correlation was run to correlate FSQ (fear of spiders) and STAI-trait (trait anxiety) scores with the mean MSE values across scales 1 to 10 of each ROI on all subjects from either group (corrplot package v 0.94). The p-values of the 24 correlations were FDR corrected. For clarity purposes, the complete correlation matrices between the MSE of the 12 ROIs and the FSQ and STAItrait were additionally computed for each scale and separated by group (pheatmap package v 1.0.13).

### 2.6. 7T MSE validation and comparability

As the introduction outlines, the higher field strength of 7T fMRI leads to a higher tSNR than 3T. At the same time, the higher magnetic field strength induced a higher susceptibility to physiological artifacts and signal distortions. Small subcortical brain structures, such as the BNST or nuclei with high levels of iron, might add to the difficulty of acquiring a valid signal (de Hollander et al., 2017). Therefore, it is crucial to examine the validity of the fMRI timeseries signal in the ROIs of this study. For this purpose, the voxel-wise (VW) tSNR of the smoothed data were calculated by dividing mean intensity of the 360 datapoints of the resting-state fMRI time series by the standard deviation of the intensity: $tSNR_{VW}=\frac{Mean_{\text{signal intensity}}}{SD_{\text{signal intensity}}}$. The tSNR_VW_ was averaged over all voxels within each participant’s ROI.

To explore whether regional MSE alterations of SP derived from 7T data are as sensitive as a functional connectivity measure, we performed an intrinsic connectivity (IC) analysis (Denier et al., 2024; Martuzzi et al., 2011). IC was defined as the mean square of correlation coefficients (*r*) between each single grey matter voxel (*x*) and all other grey matter voxels (*y*) within the brain (*B*): $\text{IC}(x)={\left(\int_{y}^{B}r^{2}(x,y)dy\right)}^{1/2}$ This measure includes the number of connections and the connectivity strengths. All IC values of the voxels within each of the 12 ROIs were averaged, and analogous to the MSE ANCOVA, a 2 × 12 ANCOVA with group (HC/SP) as the between-subject factor and ROI (12 ROIs) as the within-subject factor, respectively, was conducted. Age and sex served as covariates, and Mauchly’s test for sphericity was used to indicate whether the resulting p-values needed Greenhouse-Geisser correction.

## 3. Results

### 3.1. Demographics and fear metrics

The study sample characteristics and statistics are depicted in Table 1. Datasets of two participants from the SP group were discarded due to widespread and partly exceptionally low MSE values. Supplementary Figure S1 highlights the two outliers relative to the remaining sample. These two subjects showed MSE values lower than those in previous studies with comparable methods (Smith et al., 2014). The group comparisons confirm higher fear of spiders and trait anxiety scores in the SP compared to the HC group. Besides, the sex ratio differed between the groups, with more women and fewer men in the SP than in the HC group. This observation reflects the higher prevalence of specific phobias in women and underpins the necessity to include the sex variable as a covariate in the analyses (Bourdon et al., 1988). With a p-threshold exceeding 0.05, the remaining variables yielded no substantial group differences. There was no group difference in global MSE, thus confirming our assumption. Lastly, comparable mean FD values demonstrated that the MSE group effects were not confounded by subject motion during data acquisition.

### 3.2. Complexity analysis

Mauchly’s test for sphericity was significant for all investigated main effects and interactions (Supplementary Table S1). Therefore, only Greenhouse-Geisser corrected statistics are reported. The mean MSE ANCOVA yielded a significant three-way interaction of group × ROI × scale (F_99_ = 1.71, *p* = 1.71e-05, η^2^ = 0.024). Besides, there was a two-way interaction of ROI × Scale (F_99_ = 7.86, *p* = 4.22e-99, η^2^ = 0.102) and a main effect of scale (F_1.3_ = 21.50, *p* = 1.66e-06, η^2^ = 0.238). None of the remaining main effects or interactions were significant (Supplementary Table S2 for the complete statistics). The covariates age and sex did not alter the findings significantly, as merely sex interacted with ROI × Scale (F_99_ = 1.50, *p* = 0.001, η^2^ = 0.021). However, as reported above, this interaction remained significant even after correcting for the covariates. The three-way interaction indicates that MSE between SP and HC, depending on the brain region and the frequency scales, is altered.

**Fig. 2.**
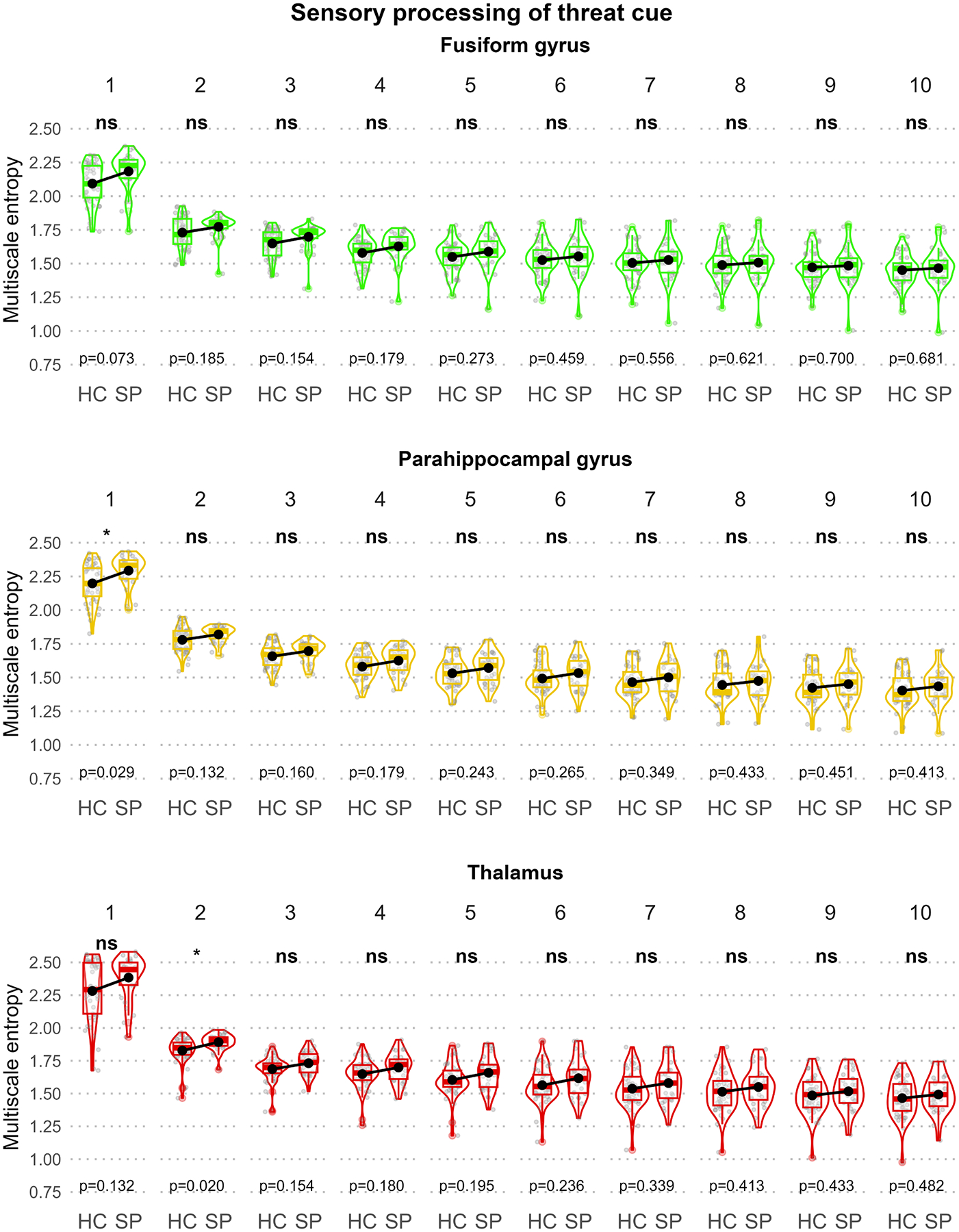
Group comparison of scale-wise regional MSE for the “Sensory processing of threat cue” unit. Box and violin plots of each panel illustrate the regional mean MSE stratified by group and scale (1 – 10). Black dots represent group means. For each scale, significant MSE group differences are indicated using asterisks at the top of the plots and p-values of the post-hoc *t*-tests at the bottom of the plots.

**Fig. 3.**
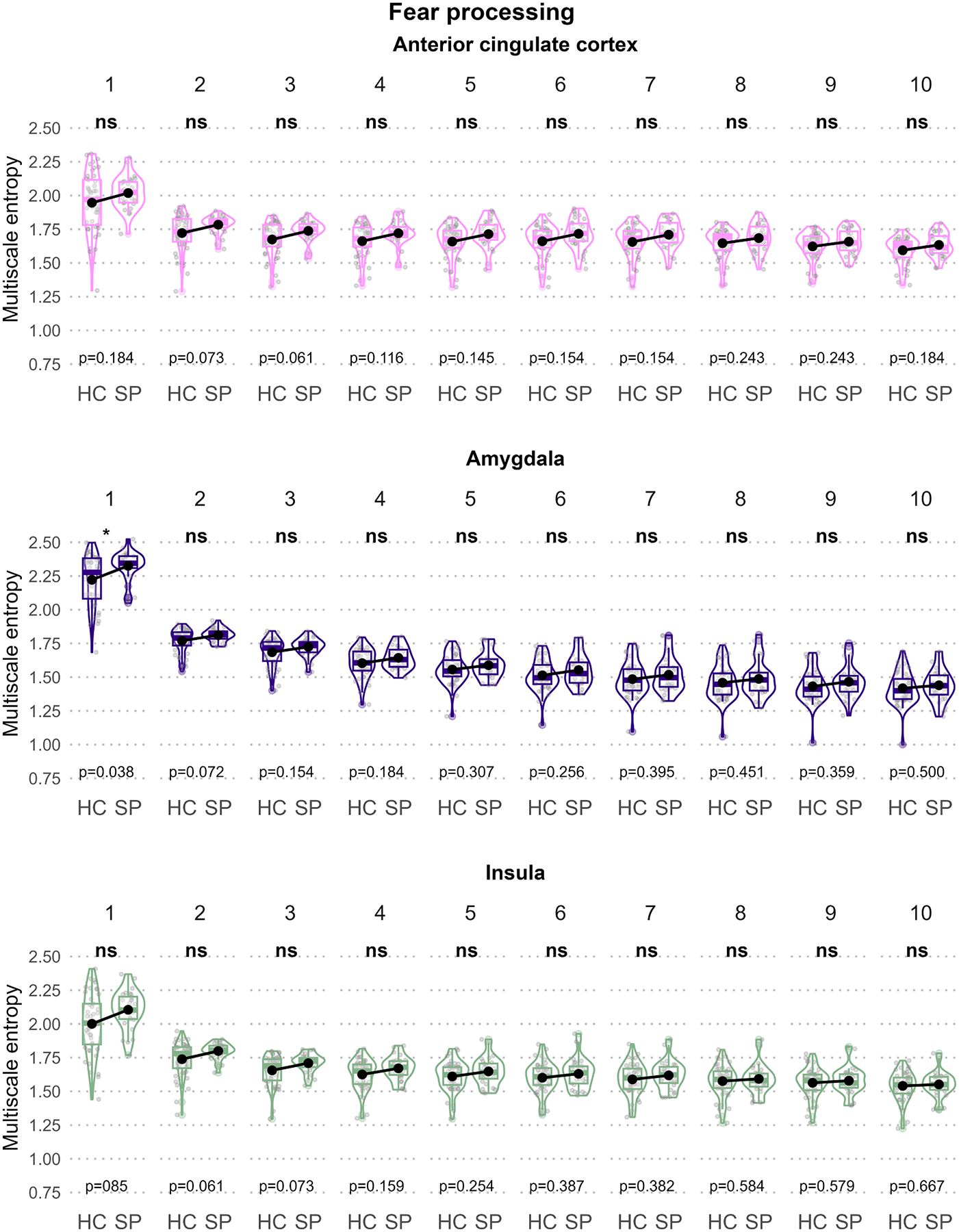
Group comparison of scale-wise regional MSE for the “Fear processing” unit. Box and violin plots of each panel illustrate the regional mean MSE stratified by group and scale (1 – 10). Black dots represent group means. For each scale, significant MSE group differences are indicated using asterisks at the top of the plots and p-values of the post-hoc *t*-tests at the bottom of the plots.

**Fig. 4.**
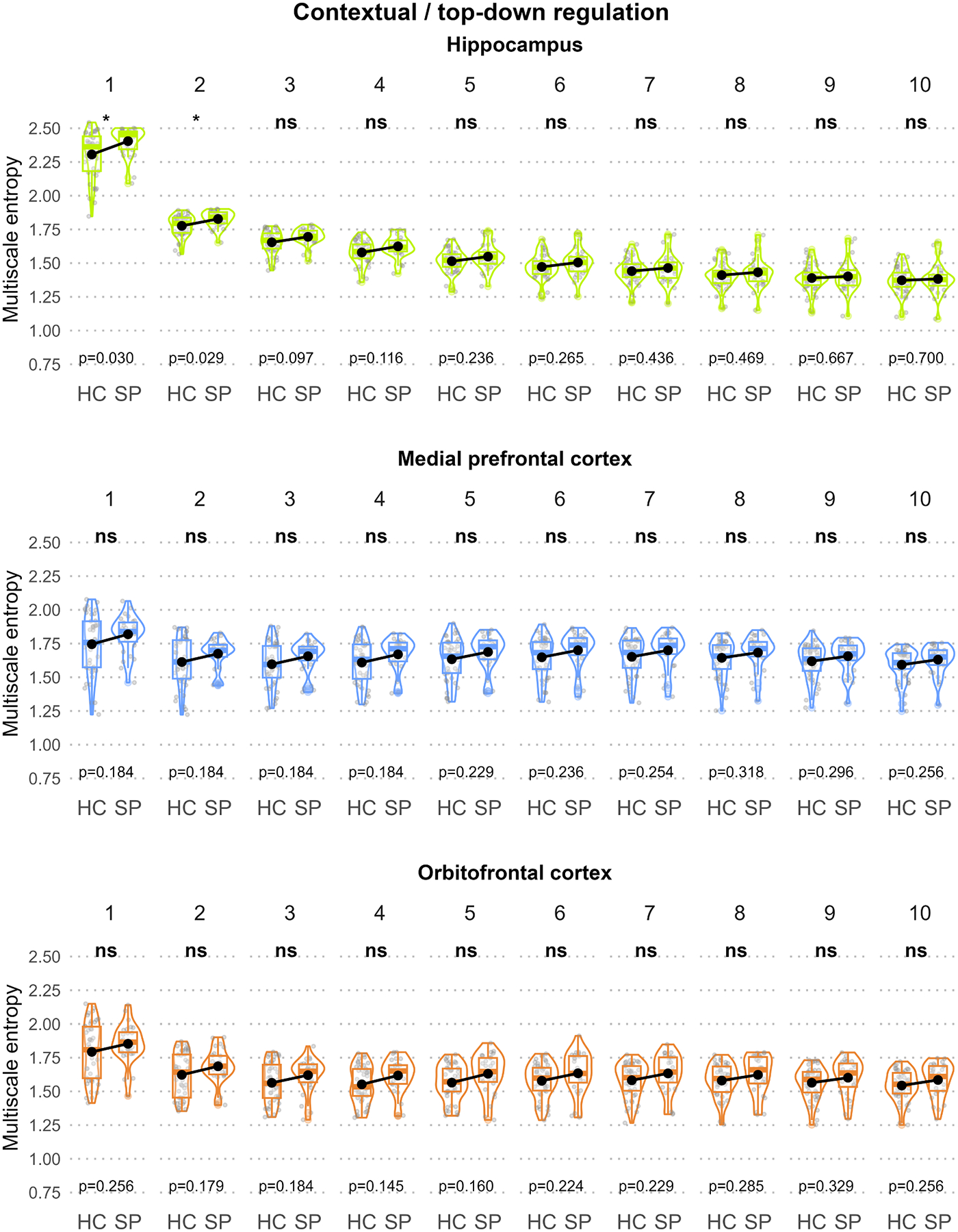
Group comparison of scale-wise regional MSE for the “contextual / top-down regulation” unit. Box and violin plots of each panel illustrate the regional mean MSE stratified by group and scale (1 – 10). Black dots represent group means. For each scale, significant MSE group differences are indicated using asterisks at the top of the plots and p-values of the post-hoc *t*-tests at the bottom of the plots.

**Fig. 5.**
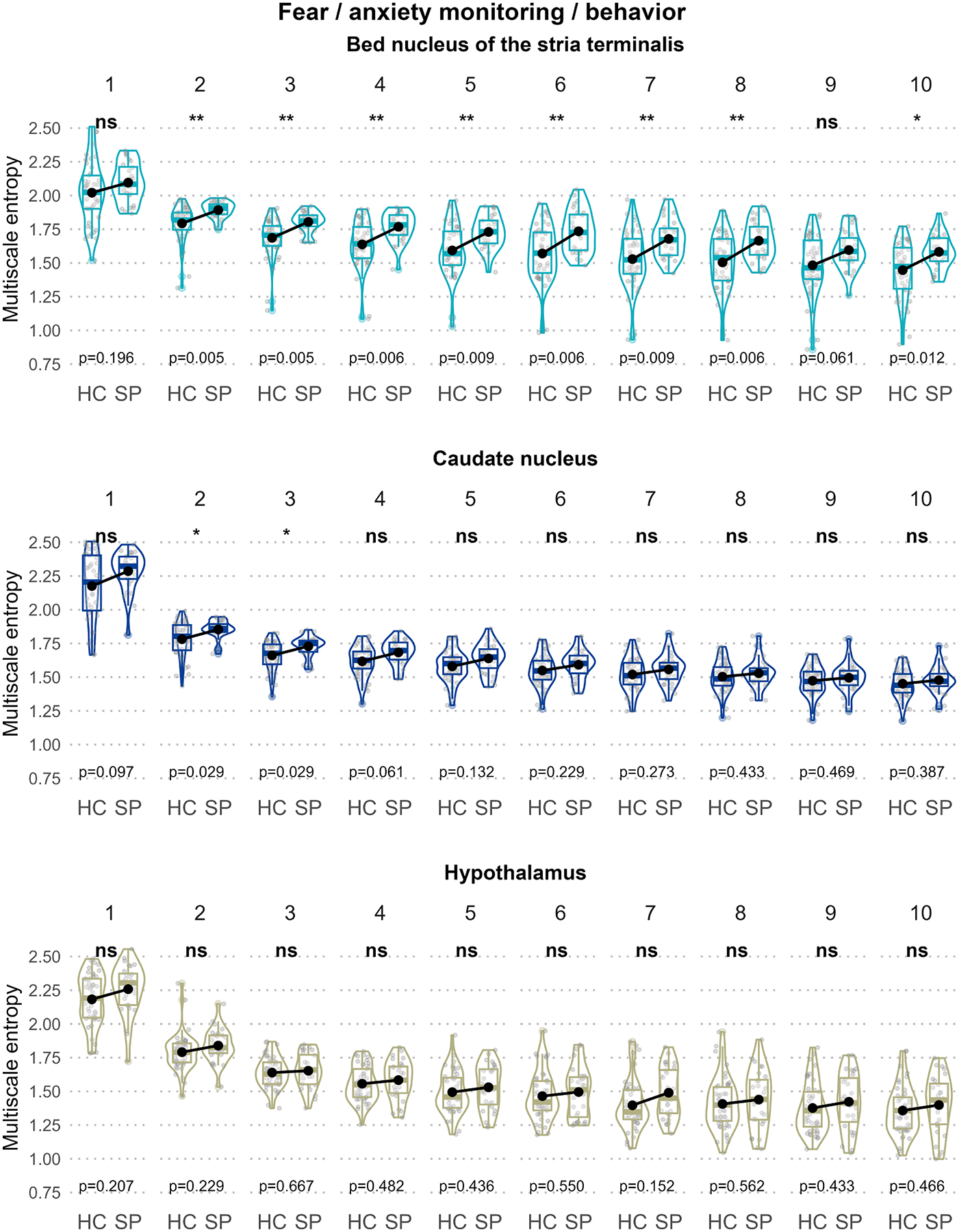
Group comparison of scale-wise regional MSE for the “fear / anxiety monitoring / behavior” unit. Box and violin plots of each panel illustrate the regional mean MSE stratified by group and scale (1 – 10). Black dots represent group means. For each scale, significant MSE group differences are indicated using asterisks at the top of the plots and p-values of the post-hoc *t*-tests at the bottom of the plots.

Figs. 2–5 illustrate the MSE for each ROI, scale, and group sorted by the predefined functional units to disentangle the three-way interaction. In the “Sensory processing of threat cue” unit (Fig. 2), increased MSE in SP compared to HC could be observed in the parahippocampal gyrus (scale 1) and the thalamus (scale 2) but not the fusiform gyrus. In the “Fear processing” unit (Fig. 3), higher MSE in SP was found in the amygdala (scale 1) but not the ACC or insula. In the “Contextual / top-down regulation” unit (Fig. 4), increased MSE in SP could be identified in the hippocampus (scales 1 and 2) but not the OFC or the MPFC. Finally, in the “Fear / anxiety monitoring / behavior” unit (Fig. 5), elevated MSE in SP was observed in the BNST (scales 2 – 8, and 10) and the caudate nucleus (scales 1 and 2) but not the hypothalamus.

### 3.3. Relationship between complexity and fear and anxiety

**Fig. 6.**
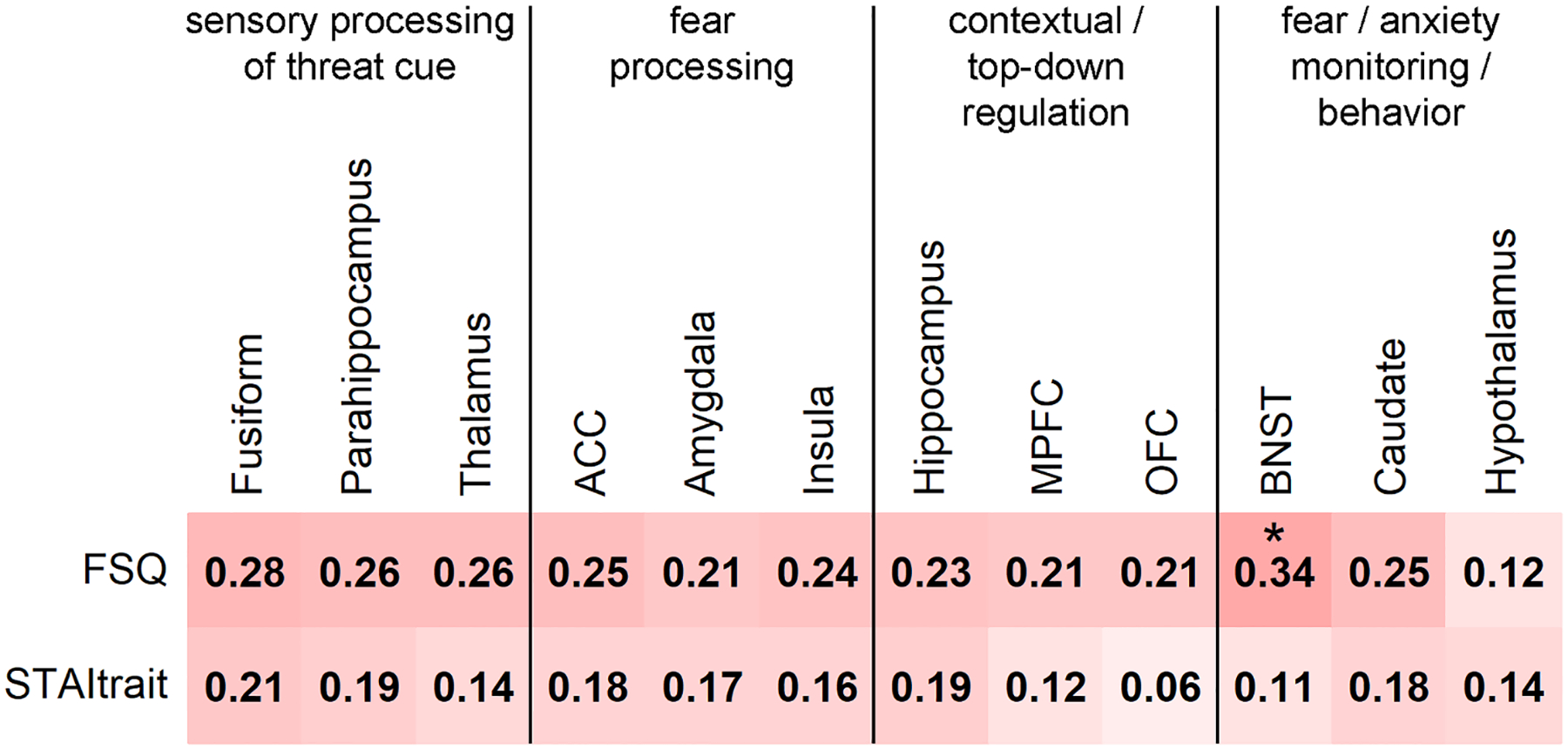
Relationship between regional MSE and spider phobic fear and trait anxiety. Spearman correlations including all study participants (SP and HC). The significant correlation is marked with an asterisk (p-values: * < 0.05). ACC: anterior cingulate cortex; BNST: bed nucleus of the stria terminalis; FSQ: Fear of Spiders Questionnaire; MPFC: medial prefrontal cortex; OFC: orbitofrontal cortex; STAItrait: State-Trait Anxiety Inventory.

The Spearman correlation analyses assessed whether regional complexity was related to spider-phobic fear or trait anxiety. Spider-phobic fear (as indexed by a high FSQ score) was positively associated with MSE in all ROIs, yet only significantly in the BNST. Spearman’s rho values between regional MSE and trait anxiety (i.e., STAI-trait) were lower than for the FSQ, with none reaching significance (Fig. 6). For completeness, in Supplementary Figure S2, the full correlation matrices between regional MSE and FSQ/STAItrait for each scale and group are illustrated. However, as discussed in the limitations section, this study did not have sufficient statistical power to test for significant group effects in the 480 correlations.

### 3.4. 7T MSE validation and comparability

Supplementary Table S3 lists the tSNR for each ROI. Generally, all ROIs apart from the hypothalamus exceeded a tSNR of 50 and appeared comparable with those of Yoo et al. (2018), based on the same TR and a similar smoothing Kernel. The hypothalamus exhibited the lowest tSNR amongst the ROIs with 33.63, which is higher than those reported in an earlier 3T study (Manuel et al., 2023). The ROI with the highest expected iron levels, the caudate nucleus, exhibited a tSNR of 69.22. The validation analysis confirmed that the fMRI timeseries used for the MSE analysis showed a sufficiently high tSNR.

The ANCOVA investigating regional IC differences between HC and SP yielded main effects of sex (F_69_ = 6.767, *p* = 0.011, η^2^ = 0.089) and ROI (F_370.5_ = 6.032, *p* = 1.26e-05, η^2^ = 0.080), but not of group, and no interactions whatsoever (see Supplementary Tables S4 and S5). Hence, no post-hoc tests were performed to investigate ROI-specific group effects. For the sake of clarity, group-specific IC for each ROI can be viewed in Supplementary Figure S3.

## 4. Discussion

### 4.1. Main findings

This proof-of-concept study is the first to introduce the domain of resting-state fMRI complexity to ultra-high field imaging with 7T in SP. Key insights are yielded that complement previous analyses using FC measures by exploring altered complex neural systems, underlying anxiety disorders in particular (e.g., Jehli et al., 2024).

The current study revealed elevated MSE complexity in SP, compared to HC, in key regions for fear and anxiety. The regional MSE augmentation varied across scales, and this frequency-specific alteration was dissimilar among some of the ROIs. After correcting for multiple testing, the BNST proved to be the only ROI where the MSE in SP was elevated in all scales, except scales 1 and 9. Additionally, lower frequency MSE (scales 4 to 10, except 9) was increased in SP but only in the BNST. Most ROIs with a group effect showed the MSE increase mainly in the lower scales 1 to 3, and thus, the higher frequencies. It should also be mentioned that no MSE difference between SP and HC was present in the fear and anxiety system when all ROIs were concatenated or in the global grey matter MSE. Finally, higher regional MSE was moderately associated with increased spider-phobic fear in most of the ROIs, yet only the BNST correlation was significant after correction for multiple testing. Such associations were much weaker with trait anxiety and were not significant.

Taken together, these results might mirror a hypersensitivity in the sensory and fear-processing regions that results in inappropriate information generation of potentially incoming phobic stimuli. Possibly, the BNST plays an important role in linking altered fear processing to increased baseline stress via its connections to hormonal structures (Lebow and Chen, 2016).

### 4.2. In-depth findings

Significant group differences were detected on at least one ROI of each predefined functional unit. This might indicate that the adaptability of the fear and anxiety circuits is elevated across the various stages in threat cue processing, monitoring and regulating. In contrast, most of the 120 group comparisons were insignificant, and 22 group differences did not survive FDR correction. Thus, almost half of the ROIs were not affected by MSE alterations linked to SP, or the statistical power was insufficient to detect such effects. Considering that the data of this study was acquired during the participants at rest, without exposure to any threat cue, the discovery of increased MSE in SP on spatial (i.e., ROI) and temporal dimensions (i.e., scale) merits a thorough inspection – potentially indicative of altered functioning in SP in supposedly benign situations and irrespective of the presence of threat-related material.

#### 4.2.1. Sensory processing of threat cue unit

Fig. 2 exemplifies that in SP MSE was elevated on the two highest frequency scales (1 Hz and 0.5 Hz) in the “sensory processing of threat cue” unit. This heightened complexity in the intra-regional processing frequencies might be linked to an altered functional architecture at the early processing stage of threat cues. At this stage, sensory signals are processed and modulated by threat anticipation (parahippocampal gyrus) and the integration of sensory input (thalamus, Maren, 2001).

This is thus in line with previous evidence showing that personal expectations affect the sensory perception of possible threats in SP (for an overview, see Aue and Okon-Singer, 2015). These findings in the sensory unit further demonstrate that the construct validity of the MSE metric might not be limited to higher-order cognition but, as the term implies, also measures more fine-grained alterations in complex neural systems.

#### 4.2.2. Fear processing unit

The fear processing unit revealed a single MSE increase for SP, relative to HC, in the amygdala on scale 1 (1 Hz). The amygdala is a key hyperactive region in specific phobia during threat exposure (Goossens et al., 2007; Stein et al., 2007b). The amygdala is not only pivotal in fear processing, but is also functionally connected to the BNST, which mediates sustained anxiety and plays a role in stress processing (Avery et al., 2014; Kim et al., 2013). The connections between the amygdala and BNST and their complex functional relationship might therefore contribute to the altered functional architecture in these fear and anxiety hubs manifested in changed MSE.

#### 4.2.3. Contextual/top-down regulation unit

Regarding top-down regulation of the apprehensive state or putting an anticipated threat cue into an anxiolytic context, the hippocampus was the only ROI showing increased MSE within this functional unit. The role of the hippocampus in SP has been attributed to fear learning and modulation, defensive reactivity during a threat, and fear extinction, which might be reflected in these MSE alterations (Lueken et al., 2014; Paquette et al., 2003). At the same time, frontal cortex regions were not affected by MSE alterations in the assessed frequency spectrum of our data, covering intra- rather than inter-regional processes. This may explain why we did not find any MSE alterations attributed to ineffective prefrontal down-regulation of excessive fear – they might rely on more distant connections (Stein et al., 2007a). Thus, although the hippocampus was assigned to the same functional unit as the MPFC and OFC, the results underscore the distinct regulatory roles of medial temporal and frontal lobe regions.

#### 4.2.4. Fear / anxiety monitoring/ behavior unit

The most robust and scale-overarching changes in MSE were found in the BNST (scales 2 – 8 and 10). Specifically, the BNST was the only ROI that showed group differences in frequency scales lower than 3 (0.25 Hz, 0.2 Hz, 0.167 Hz, 0.143 Hz, 0.125 Hz, and 0.1 Hz). By contrast, on scale 1, the highest assessed frequency (1 Hz), no group difference was found in the BNST. Note that scale 1 is a special case of the scales examined in this study, as it reflects the original signal frequency without coarse-graining and thus exhibits the greatest distance from all the remaining scales. The exact mechanism underlying the effect on MSE in the BNST has yet to be investigated. It ought to be noted in that respect that a sufficient understanding of the implications of fine-grained entropy differences between the frequencies is still lacking.

Within this functional unit, SP MSE elevations were also detected on scales 2 and 3 in the caudate nucleus, but none in the hypothalamus. Thus, the most substantial MSE increase in SP appears to be linked to threat monitoring and controlling fear and anxiety-related behaviors, given their role in the BNST and the caudate nucleus. The novel finding of the BNST playing a substantial role in SP corroborates results from an earlier study of ours where we implicated the BNST in FC alterations (Jehli et al., 2024). The caudate nucleus has functional and structural connections to the BNST, and they share a threat-monitoring function (Avery et al., 2014; Mobbs et al., 2010) – as a subregion of the striatum, it is also involved in threat anticipation (Drabant et al., 2011).

#### 4.2.5. Correlations with fear and anxiety

In line with the regional MSE alterations in SP, we found a positive association between MSE and spider-phobic fear, but not between MSE and trait anxiety. This fear-related association was only significant in the BNST after FDR correction. One should nonetheless consider that correlations were computed using fear and anxiety scores with mean MSE values over all scales and across the total study sample. This approach might have contributed to comparably small effect sizes (i.e. moderate rho values). Principally, this was due to including scales, without group difference effects or with negative rho values – i.e., they were inversely linked to SP or rendered irrelevant. In our view, the stronger relationship between MSE and spider-phobic fear severity relative to trait anxiety underlines the pertinence of MSE alterations to our understanding of SP.

### 4.3. Synthesis

The increased MSE in brain regions of the fear and anxiety circuitry (e.g., amygdala, hippocampus, and parahippocampal gyrus) is in line with the notion of hyperactivity in these regions in SP (see Soravia et al., 2016). Indeed, the elevated complexity found in this study might reflect an inherent hypersensitivity to adapt the fear and anxiety system to potentially threatening situations that are, objectively, not that endangering. This hypersensitivity or reactivity in the context of MSE increments paired with anxiety as a quasi-stable mental condition can be seen as a hyperresponsive state, heightening alertness and response to fearful stimuli. This idea is supported by animal research showing that complexity of local field potentials and of the BOLD signal decreases significantly when altering the excitation/inhibition (E/I) balance (Wang et al., 2018). Critically, increases in the E/I balance will likely manifest as increases in complexity, as observed in this study. The assumed apprehensive state in SP is termed paranoia in schizophrenia spectrum disorders, and has been related to increased FC between the amygdala and hippocampus in prior work (Walther et al., 2022); both of these regions exhibited increased MSE in SP in this study. These MSE outcomes tie in with results from Jehli et al. (2024) in virtually the same sample, showing increased FC in the amygdala in SP with elevated phobic fear. Interestingly, this study also yielded changes in FC in the BNST, specifically in association with general anxiety and social anxiety. It seems that the consistent occurrence of BNST alterations in both MSE and FC is linked to the higher spatial specificity of 7T MRI compared to 3T. Notably, the FC results were based on correlations with clinical scales, but neither FC nor IC showed any significant group differences. This null effect of group confirms our assumption that the MSE measure is more sensitive to subtle neural changes than FC or IC metrics.

Other research on MSE alterations in mental disorders has primarily found reductions in entropy. The comparison of the diseases examined in those works with SP appears delicate, as they were either degenerative (Grieder et al., 2018; Jann et al., 2023) or in younger cohorts with a different symptomatology (Zhang et al., 2024). Increased brain entropy has, in turn, been found in cohorts with obsessive-compulsive disorder (OCD, Jiang et al., 2021) and generalized anxiety disorder (Fan et al., 2023). It is thus possible that elevated brain entropy is found in disorders characterized by interpersonal sensitivity and obsessive doubts, features common to both SP and OCD (Carpita et al., 2020). Taken together, our finding of SP-related MSE augmentation across several frequencies and brain regions adds critical insight into the temporal dynamics of neural processing complexity in mental disorders.

### 4.4. Limitations

A limitation of the present study is the unequal sex distribution between SP and HC. Since sex differences may influence fear-related neural processing, this imbalance could have introduced a confounding effect. To mitigate this, we statistically controlled for sex in our group-level analyses. Nevertheless, future studies should aim to recruit sex-matched groups to disentangle sex-specific contributions to fear processing further. The limited frequency range covered by the scanning protocol may constitute another limitation of this study. With a total acquisition time of six minutes and a TR of 1000 ms, the lowest reliable frequency included in the analyses was 0.1 Hz (with 36 data points), which is considered the lower limit of the high-frequency range (Wang et al., 2018). Our study, therefore, comprised only high but not low frequencies; longer acquisition times are needed to address this issue. However, one could hold that frequencies between 0.001 and 0.1 Hz are already covered by most studies assessing FC metrics. The higher frequencies, in turn, have received comparatively less attention, although they are also linked to FC (Cordes et al., 2001; Smith et al., 2013). Inspecting Figs. 2–5, MSE group differences across ROIs and scales appear relatively small. Similarly, Spearman’s rho coefficients as a measure of effect size of the correlations between regional MSE and FSQ are small to moderate. A study design including 22 factorial steps concerning MSE alone inherently leads to a high number of statistical tests, requiring some form of correction for multiple testing. On the one hand, one might hold that this study had a power problem (i.e., insufficient sample size), as numerous effects did not overcome FDR correction. On the other hand, this study relied on precise and sensitive measures of the neural architecture (7T and MSE), providing sufficient power to identify novel findings – often unmatched in other research (Nebe et al., 2023). For example, with tSNR values ranging between 33 and 75 %, several group differences were detected for MSE but not IC. The fact that the correlational effect between MSE in the BNST and FSQ was computed across the entire study sample may constitute another limitation in that it might have been driven by the group differences in FSQ. Finally, the numerous inferences made regarding the specific functional roles of the included ROIs in this study could be perceived as yet another limitation. These functional roles were drawn from other studies and cannot be verified with the current dataset. Nonetheless, the current study revealed a correlational link between MSE and phobic fear severity, thereby confirming the functional relevance of these regions for SP. Task-based approaches may need to establish causal relationships between entropy measures and cognitive functions of segregated brain regions, although metrics other than MSE might be more suitable in this context (Wehrheim et al., 2024).

### 4.5. Conclusions

As demonstrated by the link between MSE and spider-phobic fear uncovered in this study, increased MSE in the fear and anxiety circuits is related to elementary hypervigilance in individuals affected by SP. MSE is a promising measure, permitting the detection of comparably small yet robust differences when combined with the higher temporal and spatial SNR of 7T fMRI, as this study advocates. Using MSE as a complementary measure of conventional FC measures, a more refined picture of the dynamics of complex neural systems can be achieved. MSE has been linked to higher-order cognition (Omidvarnia et al., 2021). Yet, the current findings underline the potential role of MSE in integrating sensory processes, subserving such higher-order cognitive functions. This suggestion is supported by the increases in MSE in the fusiform gyrus, the parahippocampal gyrus, and the thalamus in individuals with SP. Whereas the fusiform gyrus responds to visual cue configurations, thalamic processes are linked to arousal, fear, and anxiety responses (Del Casale et al., 2012; Duval et al., 2015). These processes, in turn, may provide cognitive correlates of our MSE effects in these regions and merit closer examination in dedicated future studies.

This is the first study examining MSE in 7T fMRI, capitalizing on the improved image resolution at this field strength. Nonetheless, we cannot quantify its expected superior sensitivity relative to lower magnetic field MRI, as comparable data with 3T does not exist. However, in a previous 3T study of ours on mild Alzheimer’s disease, we only detected MSE-linked group differences on two scales and in one ROI (Grieder et al. 2018). This is in stark contrast to the current study with 7T, demonstrating a larger amount of MSE differences across a multitude of scales and regions. Albeit this difference in effects may be disorder-specific, it could also be seen as a clear indication of the capability of 7T to detect more subtle changes in MSE. To conclude, this study emphasizes the potential of 7T complexity in illuminating and expanding our understanding of symptom-linked alterations in the neural architecture of anxiety disorders.

## Supplementary Material

1

Supplementary material associated with this article can be found, in the online version, at doi:10.1016/j.neuroimage.2025.121371.

## Figures and Tables

**Table 1:** Study sample demographics and statistics.

	HC (*N* = 45)	SP (*N* = 28)	HC-SP	
	Mean (SD)	Mean (SD)	*statistics*	*p*
Age, years	26.3 (6.0)	27.8 (4.6)	475.0 (*U*)	0.08
Sex, f/m	17/28	24/4	16.1 (*X*^*2*^)	**5.98e-05**
Education, years	16.1 (2.8)	15.9 (2.2)	0.37 (*T*)	0.71
FSQ, score	24.8 (9.6)	85.7 (13.9)	2.0 (*U*)	**7.81e-13**
STAItrait, score	55.5 (8.7)	60.2 (9.4)	−2.2 (*T*)	**0.03**
BSCL, score	10.6 (6.8)	16.0 (14.1)	530.5 (*U*)	0.26
Global MSE	1.64 (0.13)	1.57 (0.27)	513.0 (*U*)	0.18
Mean FD, mm	0.09 (0.03)	0.09 (0.02)	589.0 (*U*)	0.64

Note: HC: healthy controls, SP: spider-fearful participants, SD: standard deviation, FSQ: Fear of Spiders Questionnaire, BSCL: Brief Symptom Checklist, STAI-trait: State-Trait Anxiety Inventory, FD: Framewise Displacement. *p* < 0.05 is highlighted in bold.

## Data Availability

Codes used for data analysis are made available upon request to the corresponding author. The data are not publicly available due to privacy or ethical restrictions, but will be provided upon request to the corresponding author.

## Data availability

Data will be made available on request.

## References

[R1] Aue T, Okon-Singer H, 2015. Expectancy biases in fear and anxiety and their link to biases in attention. Clin. Psychol. Rev 42, 83–95. 10.1016/j.cpr.2015.08.005. 26379081

[R2] Avery SN, Clauss JA, Blackford JU, 2016. The Human BNST: functional role in anxiety and addiction. Neuropsychopharmacology 41, 126–141. 10.1038/npp.2015.185. 26105138 PMC4677124

[R3] Avery SN, Clauss JA, Winder DG, Woodward N, Heckers S, Blackford JU, 2014. BNST neurocircuitry in humans. Neuroimage 91, 311–323. 10.1016/j.neuroimage.2014.01.017. 24444996 PMC4214684

[R4] Bassett DS, Meyer-Lindenberg A, Achard S, Duke T, Bullmore E, 2006. Adaptive reconfiguration of fractal small-world human brain functional networks. Proc. Natl. Acad. Sci 103, 19518–19523. 10.1073/pnas.0606005103. 17159150 PMC1838565

[R5] Behzadi Y, Restom K, Liau J, Liu TT, 2007. A component based noise correction method (CompCor) for BOLD and perfusion based fMRI. Neuroimage 37, 90–101. 10.1016/j.neuroimage.2007.04.042. 17560126 PMC2214855

[R6] Biswal B, Yetkin FZ, Haughton VM, Hyde JS, 1995. Functional connectivity in the motor cortex of resting human brain using echo-planar MRI. Magn. Reson. Med 34, 537–541. 8524021 10.1002/mrm.1910340409

[R7] Boehme S, Ritter V, Tefikow S, Stangier U, Strauss B, Miltner WHR, Straube T, 2013. Brain activation during anticipatory anxiety in social anxiety disorder. Soc. Cogn. Affect. Neurosci 9, 1413–1418. 10.1093/scan/nst129. 23938870 PMC4158379

[R8] Bourdon KH, Boyd JH, Rae DS, Burns BJ, Thompson JW, Locke BZ, 1988. Gender differences in phobias: results of the ECA community survey. J. Anxiety Disord 2, 227–241. 10.1016/0887-6185(88)90004-7.

[R9] Buff C, Brinkmann L, Bruchmann M, Becker MPI, Tupak S, Herrmann MJ, Straube T, 2017. Activity alterations in the bed nucleus of the stria terminalis and amygdala during threat anticipation in generalized anxiety disorder. Soc. Cogn. Affect. Neurosci 12, 1766–1774. 10.1093/scan/nsx103. 28981839 PMC5714227

[R10] Calhoun VD, Adali T, 2012. Multisubject independent component analysis of fMRI: a decade of intrinsic networks, default mode, and neurodiagnostic discovery. IEEE Rev. Biomed. Eng 5, 60–73. 10.1109/RBME.2012.2211076. 23231989 PMC4433055

[R11] Carpita B, Muti D, Petrucci A, Romeo F, Gesi C, Marazziti D, Carmassi C, Dell’Osso L, 2020. Overlapping features between social anxiety and obsessive-compulsive spectrum in a clinical sample and in healthy controls: toward an integrative model. CNS Spectr. 25, 527–534. 10.1017/s109285291900138x. 31576793

[R12] Colizoli O, de Gee JW, van der Zwaag W, Donner TH, 2020. Comparing fMRI Responses Measured at 3 Versus 7 Tesla Across Human Cortex, Striatum, and Brainstem. bioRxiv. 10.1101/2020.05.12.090175, 2020.2005.2012.090175.

[R13] Cordes D, Haughton VM, Arfanakis K, Carew JD, Turski PA, Moritz CH, Quigley MA, Meyerand ME, 2001. Frequencies contributing to functional connectivity in the Cerebral cortex in “resting-state” data. Am. J. Neuroradiol 22, 1326–1333. 11498421 PMC7975218

[R14] Costa M, Goldberger AL, Peng CK, 2002. Multiscale entropy analysis of complex physiologic time series. Phys. Rev. Lett 89, 068102. 10.1103/PhysRevLett.89.068102. 12190613

[R15] Damoiseaux JS, Rombouts SA, Barkhof F, Scheltens P, Stam CJ, Smith SM, Beckmann CF, 2006. Consistent resting-state networks across healthy subjects. Proc. Natl. Acad. Sci. USA 103, 13848–13853. 10.1073/pnas.0601417103. 16945915 PMC1564249

[R16] Davis M, Walker DL, Miles L, Grillon C, 2010. Phasic vs sustained fear in rats and humans: role of the extended amygdala in fear vs anxiety. Neuropsychopharmacology 35, 105–135. 10.1038/npp.2009.109. 19693004 PMC2795099

[R17] de Hollander G, Keuken MC, van der Zwaag W, Forstmann BU, Trampel R, 2017. Comparing functional MRI protocols for small, iron-rich basal ganglia nuclei such as the subthalamic nucleus at 7 T and 3 T. Hum. Brain Mapp 38, 3226–3248. 10.1002/hbm.23586. 28345164 PMC6867009

[R18] de Quervain DJF, Margraf J, 2008. Glucocorticoids for the treatment of post-traumatic stress disorder and phobias: a novel therapeutic approach. Eur. J. Pharmacol 583, 365–371. 10.1016/j.ejphar.2007.11.068. 18275950

[R19] Del Casale A, Ferracuti S, Rapinesi C, Serata D, Piccirilli M, Savoja V, Kotzalidis GD, Manfredi G, Angeletti G, Tatarelli R, Girardi P, 2012. Functional neuroimaging in specific phobia. Psychiatry Res.: Neuroimaging 202, 181–197. 10.1016/j.pscychresns.2011.10.009. 22804970

[R20] Denier N, Grieder M, Jann K, Breit S, Mertse N, Walther S, Soravia LM, Meyer A, Federspiel A, Wiest R, Bracht T, 2024. Analyzing fractal dimension in electroconvulsive therapy: unraveling complexity in structural and functional neuroimaging. Neuroimage, 120671. 10.1016/j.neuroimage.2024.120671. 38901774

[R21] Drabant EM, Kuo JR, Ramel W, Blechert J, Edge MD, Cooper JR, Goldin PR, Hariri AR, Gross JJ, 2011. Experiential, autonomic, and neural responses during threat anticipation vary as a function of threat intensity and neuroticism. Neuroimage 55, 401–410. 10.1016/j.neuroimage.2010.11.040. 21093595 PMC3031673

[R22] Dumoulin SO, Fracasso A, van der Zwaag W, Siero JCW, Petridou N, 2018. Ultra-high field MRI: advancing systems neuroscience towards mesoscopic human brain function. Neuroimage 168, 345–357. 10.1016/j.neuroimage.2017.01.028. 28093360

[R23] Duval ER, Javanbakht A, Liberzon I, 2015. Neural circuits in anxiety and stress disorders: a focused review. Ther. Clin. Risk Manag 11, 115–126. 10.2147/TCRM.S48528. 25670901 PMC4315464

[R24] Fan S, Yu Y, Wu Y, Kai Y, Wang H, Chen Y, Zu M, Pang X, Tian Y, 2023. Altered brain entropy and functional connectivity patterns in generalized anxiety disorder patients. J. Affect. Disord 332, 168–175. 10.1016/j.jad.2023.03.062. 36972849

[R25] Fernández A, Gómez C, Hornero R, López-Ibor JJ, 2013. Complexity and schizophrenia. Prog. Neuro-Psychopharmacol. Biol. Psychiatry 45, 267–276. 10.1016/j.pnpbp.2012.03.015. 22507763

[R26] Franke GH, 2017. Brief Symptom Checklist (BSCL). Manual. Hogrefe-Verlag, Göttingen

[R27] Goltermann J, Winter NR, Meinert S, Sindermann L, Lemke H, Leehr EJ, Grotegerd D, Winter A, Thiel K, Waltemate L, Breuer F, Repple J, Gruber M, Richter M, Teckentrup V, Kroemer NB, Brosch K, Meller T, Pfarr J-K, Ringwald KG, Stein F, Heindel W, Jansen A, Kircher T, Nenadić I, Dannlowski U, Opel N, Hahn T, 2023. Resting-state functional connectivity patterns associated with childhood maltreatment in a large bicentric cohort of adults with and without major depression. Psychol. Med 53, 4720–4731. 10.1017/S0033291722001623. 35754405 PMC10388325

[R28] Goossens L, Sunaert S, Peeters R, Griez EJL, Schruers KRJ, 2007. Amygdala hyperfunction in phobic fear normalizes after exposure. Biol. Psychiatry 62, 1119–1125. 10.1016/j.biopsych.2007.04.024. 17706612

[R29] Grieder M, Wang DJJ, Dierks T, Wahlund L-O, Jann K, 2018. Default mode network complexity and cognitive decline in mild Alzheimer’s disease. Front. Neurosci 12. 10.3389/fnins.2018.00770. PMC6206840 30405347

[R30] He BJ, 2011. Scale-free properties of the functional magnetic resonance imaging signal during rest and task. J. Neurosci 31, 13786–13795. 10.1523/JNEUROSCI.2111-11.2011. 21957241 PMC3197021

[R31] He BJ, Zempel JM, Snyder AZ, Raichle ME, 2010. The temporal structures and functional significance of scale-free brain activity. Neuron 66, 353–369. 10.1016/j.neuron.2010.04.020. 20471349 PMC2878725

[R32] Jann K, 2021. LOFT Complexity Toolbox. GitHub repository.

[R33] Jann K, Boudreau J, Albrecht D, Cen SY, Cabeen RP, Ringman JM, Wang DJJ, 2023. FMRI complexity correlates with tau-PET and cognitive decline in late-onset and autosomal dominant Alzheimer’s disease. J. Alzheimer’s Dis 95, 437–451. 10.3233/JAD-220851. 37599531 PMC10578217

[R34] Jann K, Kottlow M, Dierks T, Boesch C, Koenig T, 2010. Topographic electrophysiological signatures of FMRI resting state networks. PLoS One 5. 10.1371/journal.pone.0012945. PMC2943931 20877577

[R35] Jehli E, Denier N, Federspiel A, Dierks T, Strik W, Soravia LM, Grieder M, 2024. Altered functional coupling of the bed nucleus of the stria terminalis and amygdala in spider phobic fear. Brain Connect. 14, 527–541. 10.1089/brain.2024.0031. 39302065

[R36] Jiang X, Li X, Xing H, Huang X, Xu X, Li J, 2021. Brain entropy study on obsessive-compulsive disorder using resting-State fMRI. Front. Psychiatry 12. 10.3389/fpsyt.2021.764328. PMC8632866 34867549

[R37] Kim S-Y, Adhikari A, Lee SY, Marshel JH, Kim CK, Mallory CS, Lo M, Pak S, Mattis J, Lim BK, Malenka RC, Warden MR, Neve R, Tye KM, Deisseroth K, 2013. Diverging neural pathways assemble a behavioural state from separable features in anxiety. Nature 496, 219–223. 10.1038/nature12018. 23515158 PMC6690364

[R38] Lancaster JL, Woldorff MG, Parsons LM, Liotti M, Freitas CS, Rainey L, Kochunov PV, Nickerson D, Mikiten SA, Fox PT, 2000. Automated Talairach atlas labels for functional brain mapping. Hum. Brain Mapp 10, 120–131. 10.1002/1097-0193. 10912591 PMC6871915

[R39] LeBeau RT, Glenn D, Liao B, Wittchen H-U, Beesdo-Baum K, Ollendick T, Craske MG, 2010. Specific phobia: a review of DSM-IV specific phobia and preliminary recommendations for DSM-V. Depress. Anxiety 27, 148–167. 10.1002/da.20655. 20099272

[R40] Lebow MA, Chen A, 2016. Overshadowed by the amygdala: the bed nucleus of the stria terminalis emerges as key to psychiatric disorders. Mol. Psychiatry 21, 450–463. 10.1038/mp.2016.1. 26878891 PMC4804181

[R41] Leehr EJ, Seeger FR, Böhnlein J, Gathmann B, Straube T, Roesmann K, Junghöfer M, Schwarzmeier H, Siminski N, Herrmann MJ, Langhammer T, Goltermann J, Grotegerd D, Meinert S, Winter NR, Dannlowski U, Lueken U, 2024. Association between resting-state connectivity patterns in the defensive system network and treatment response in spider phobia—a replication approach. Transl. Psychiatry 14, 137. 10.1038/s41398-024-02799-x. 38453896 PMC10920691

[R42] Li X, Zhu Z, Zhao W, Sun Y, Wen D, Xie Y, Liu X, Niu H, Han Y, 2018. Decreased resting-state brain signal complexity in patients with mild cognitive impairment and Alzheimer’s disease: a multi-scale entropy analysis. Biomed. Opt. Express 9, 1916–1929. 10.1364/BOE.9.001916. 29675329 PMC5905934

[R43] Lucas A, Cornblath EJ, Sinha N, Caciagli L, Hadar P, Tranquille A, Stein JM, Das S, Davis KA, 2023. Improved Seizure Onset-Zone Lateralization in Temporal Lobe Epilepsy using 7T Resting-State fMRI: A Direct Comparison with 3T. medRxiv. 10.1101/2023.06.06.23291025. PMC12455467 40372884

[R44] Lueken U, Hilbert K, Stolyar V, Maslowski NI, Beesdo-Baum K, Wittchen H-U, 2014. Neural substrates of defensive reactivity in two subtypes of specific phobia. Soc. Cogn. Affect. Neurosci 9, 1668–1675. 10.1093/scan/nst159. 24174207 PMC4221204

[R45] Maldjian JA, Laurienti PJ, Burdette JH, 2004. Precentral gyrus discrepancy in electronic versions of the Talairach atlas. Neuroimage 21, 450–455. 10.1016/j.neuroimage.2003.09.032. 14741682

[R46] Maldjian JA, Laurienti PJ, Kraft RA, Burdette JH, 2003. An automated method for neuroanatomic and cytoarchitectonic atlas-based interrogation of fMRI data sets. Neuroimage 19, 1233–1239. 10.1016/S1053-8119(03)00169-1. 12880848

[R47] Manuel J, Halbe E, Ewald AC, Hoff A, Jordan J, Tank J, Heusser K, Gerlach DA, 2023. Glucose-sensitive hypothalamic nuclei traced through functional magnetic resonance imaging. Front. Neurosci 17. 10.3389/fnins.2023.1297197, 2023. PMC10749345 38146542

[R48] Maren S, 2001. Neurobiology of Pavlovian fear conditioning. Annu. Rev. Neurosci 24, 897–931. 10.1146/annurev.neuro.24.1.897. 11520922

[R49] Martuzzi R, Ramani R, Qiu M, Shen X, Papademetris X, Constable RT, 2011. A whole-brain voxel based measure of intrinsic connectivity contrast reveals local changes in tissue connectivity with anesthetic without a priori assumptions on thresholds or regions of interest. Neuroimage 58, 1044–1050. 10.1016/j.neuroimage.2011.06.075. 21763437 PMC3183817

[R50] McDonough IM, Nashiro K, 2014. Network complexity as a measure of information processing across resting-state networks: evidence from the Human Connectome Project. Front. Hum. Neurosci 8. 10.3389/fnhum.2014.00409. PMC4051265 24959130

[R51] Medel V, Irani M, Crossley N, Ossandón T, Boncompte G, 2023. Complexity and 1/f slope jointly reflect brain states. Sci. Rep 13, 21700. 10.1038/s41598-023-47316-0. 38065976 PMC10709649

[R52] Mobbs D, Yu R, Rowe JB, Eich H, FeldmanHall O, Dalgleish T, 2010. Neural activity associated with monitoring the oscillating threat value of a tarantula. Proc. Natl. Acad. Sci 107, 20582–20586. 10.1073/pnas.1009076107. 21059963 PMC2996708

[R53] Münsterkötter AL, Notzon S, Redlich R, Grotegerd D, Dohm K, Arolt V, Kugel H, Zwanzger P, Dannlowski U, 2015. Spider or No Spider? Neural correlates of sustained and phasic fear in Spider phobia. Depress. Anxiety 32, 656–663. 10.1002/da.22382. 26115440

[R54] Nakataki M, Soravia LM, Schwab S, Horn H, Dierks T, Strik W, Wiest R, Heinrichs M, de Quervain DJF, Federspiel A, Morishima Y, 2017. Glucocorticoid administration improves aberrant fear-processing networks in spider phobia. Neuropsychopharmacology 42, 485–494. 10.1038/npp.2016.207. 27644128 PMC5399241

[R55] Nebe S, Reutter M, Baker DH, Bölte J, Domes G, Gamer M, Gärtner A, Gießing C, Gurr C, Hilger K, Jawinski P, Kulke L, Lischke A, Markett S, Meier M, Merz CJ, Popov T, Puhlmann LMC, Quintana DS, Schäfer T, Schubert A-L, Sperl MFJ, Vehlen A, Lonsdorf TB, Feld GB, 2023. Enhancing precision in human neuroscience. eLife 12, e85980. 10.7554/eLife.85980. 37555830 PMC10411974

[R56] Nieto-Castanon A, & Whitfield-Gabrieli S, 2021. CONN functional connectivity toolbox (RRID:SCR_009550) version 21. 10.56441/hilbertpress.2161.7292. 22642651

[R57] Northoff G, Wainio-Theberge S, Evers K, 2020. Is temporo-spatial dynamics the “common currency” of brain and mind? In quest of “Spatiotemporal Neuroscience”. Phys. Life Rev 33, 34–54. 10.1016/j.plrev.2019.05.002. 31221604

[R58] S. Omidvarnia A, Zalesky A, Mansour L, Van De Ville D, Jackson GD, Pedersen M, 2021. Temporal complexity of fMRI is reproducible and correlates with higher order cognition Neuroimage 230, 117760. 10.1016/j.neuroimage.2021.117760. 33486124

[R59] Paquette V, Lévesque J, Mensour B, Leroux J-M, Beaudoin G, Bourgouin P, Beauregard M, 2003. Change the mind and you change the brain”: effects of cognitive-behavioral therapy on the neural correlates of spider phobia. Neuroimage 18, 401–409. 10.1016/S1053-8119(02)00030-7. 12595193

[R60] Pincus SM, 1991. Approximate entropy as a measure of system complexity. Proc. Natl. Acad. Sci 88, 2297–2301. 10.1073/pnas.88.6.2297. 11607165 PMC51218

[R61] Pohmann R, Speck O, Scheffler K, 2016. Signal-to-noise ratio and MR tissue parameters in human brain imaging at 3, 7, and 9.4 tesla using current receive coil arrays. Magn. Reson. Med 75, 801–809. 10.1002/mrm.25677. 25820458

[R62] Power JD, Barnes KA, Snyder AZ, Schlaggar BL, Petersen SE, 2012. Spurious but systematic correlations in functional connectivity MRI networks arise from subject motion. Neuroimage 59, 2142–2154. 10.1016/j.neuroimage.2011.10.018. 22019881 PMC3254728

[R63] Raichle ME, MacLeod AM, Snyder AZ, Powers WJ, Gusnard DA, Shulman GL, 2001. A default mode of brain function. Proc. Natl. Acad. Sci. USA 98, 676–682. 10.1073/pnas.98.2.676. 11209064 PMC14647

[R64] Schweckendiek J, Klucken T, Merz CJ, Tabbert K, Walter B, Ambach W, Vaitl D, Stark R, 2011. Weaving the (neuronal) web: fear learning in spider phobia. Neuroimage 54, 681–688. 10.1016/j.neuroimage.2010.07.049. 20673801

[R65] Smith RX, Yan L, Wang DJJ, 2014. Multiple time scale complexity analysis of resting state FMRI. Brain Imaging Behav. 8, 284–291. 10.1007/s11682-013-9276-6. 24242271 PMC4011934

[R66] Smith SM, Vidaurre D, Beckmann CF, Glasser MF, Jenkinson M, Miller KL, Nichols TE, Robinson EC, Salimi-Khorshidi G, Woolrich MW, Barch DM, Uğurbil K, Van Essen DC, 2013. Functional connectomics from resting-state fMRI. Trends Cogn. Sci 17, 666–682. 10.1016/j.tics.2013.09.016. 24238796 PMC4004765

[R67] Sokunbi MO, 2014. Sample entropy reveals high discriminative power between young and elderly adults in short fMRI data sets. Front. Neuroinformatics 8. 10.3389/fninf.2014.00069. PMC4107942 25100988

[R68] Soravia LM, Orosz A, Schwab S, Nakataki M, Wiest R, Federspiel A, 2016. CBT reduces CBF: cognitive-behavioral therapy reduces cerebral blood flow in fear-relevant brain regions in spider phobia. Brain Behav. 6, e00510. 10.1002/brb3.510. 27688940 PMC5036433

[R69] Spielberger CD, 1989. State-Trait Anxiety Inventory: Bibliography, 2nd Edition ed. Consulting Psychologists Press, Palo Alto.

[R70] Stein JL, Wiedholz LM, Bassett DS, Weinberger DR, Zink CF, Mattay VS, Meyer-Lindenberg A, 2007a. A validated network of effective amygdala connectivity. Neuroimage 36, 736–745. 10.1016/j.neuroimage.2007.03.022. 17475514

[R71] Stein MB, Simmons AN, Feinstein JS, Paulus MP, 2007b. Increased amygdala and insula activation during emotion processing in anxiety-prone subjects. Am. J. Psychiatry 164, 318–327. 10.1176/ajp.2007.164.2.318. 17267796

[R72] Strik W, Stegmayer K, Walther S, Dierks T, 2017. Systems neuroscience of psychosis: mapping schizophrenia symptoms onto brain systems. Neuropsychobiology 75, 100–116. 10.1159/000485221. 29258073

[R73] Szymanski J, O’Donohue W, 1995. Fear of spiders questionnaire. J. Behav. Ther. Exp. Psychiatry 26, 31–34. 10.1016/0005-7916(94)00072-T. 7642758

[R74] Takahashi T, 2013. Complexity of spontaneous brain activity in mental disorders. Prog. Neuro-Psychopharmacol. Biol. Psychiatry 45, 258–266. 10.1016/j.pnpbp.2012.05.001. 22579532

[R75] Tzourio-Mazoyer N, Landeau B, Papathanassiou D, Crivello F, Etard O, Delcroix N, Mazoyer B, Joliot M, 2002. Automated anatomical labeling of activations in SPM using a macroscopic anatomical parcellation of the MNI MRI single-subject brain. Neuroimage 15, 273–289. 10.1006/nimg.2001.0978. 11771995

[R76] Uludağ K, Müller-Bierl B, Uğurbil K, 2009. An integrative model for neuronal activity-induced signal changes for gradient and spin echo functional imaging. Neuroimage 48, 150–165. 10.1016/j.neuroimage.2009.05.051. 19481163

[R77] Vakorin VA, Lippé S, McIntosh AR, 2011. Variability of brain signals processed locally transforms into higher connectivity with brain development. J. Neurosci 31, 6405–6413. 10.1523/jneurosci.3153-10.2011. 21525281 PMC6622682

[R78] Walther S, Lefebvre S, Conring F, Gangl N, Nadesalingam N, Alexaki D, Wüthrich F, Rüter M, Viher PV, Federspiel A, Wiest R, Stegmayer K, 2022. Limbic links to paranoia: increased resting-state functional connectivity between amygdala, hippocampus and orbitofrontal cortex in schizophrenia patients with paranoia. Eur. Arch. Psychiatry Clin. Neurosci 272, 1021–1032. 10.1007/s00406-021-01337-w. 34636951 PMC9388427

[R79] Wang DJJ, Jann K, Fan C, Qiao Y, Zang Y-F, Lu H, Yang Y, 2018. Neurophysiological basis of multi-scale entropy of brain complexity and its relationship with functional connectivity. Front. Neurosci 12. 10.3389/fnins.2018.00352. PMC5986880 29896081

[R80] Wang Z, Li Y, Childress AR, Detre JA, 2014. Brain entropy mapping using fMRI. PLoS One 9, e89948. 10.1371/journal.pone.0089948. 24657999 PMC3962327

[R81] Wehrheim MH, Faskowitz J, Schubert A-L, Fiebach CJ, 2024. Reliability of variability and complexity measures for task and task-free BOLD fMRI. Hum. Brain Mapp 45, e26778. 10.1002/hbm.26778. 38980175 PMC11232465

[R82] Yang AC, Tsai S-J, Lin C-P, Peng C-K, 2018. A strategy to reduce bias of entropy estimates in resting-state fMRI signals. Front. Neurosci 12. 10.3390/brainsci1111141510.3389/fnins.2018.00398. PMC6008384 29950971

[R83] Yoo PE, John SE, Farquharson S, Cleary JO, Wong YT, Ng A, Mulcahy CB, Grayden DB, Ordidge RJ, Opie NL, O’Brien TJ, Oxley TJ, Moffat BA, 2018. 7T-fMRI: faster temporal resolution yields optimal BOLD sensitivity for functional network imaging specifically at high spatial resolution. Neuroimage 164, 214–229. 10.1016/j.neuroimage.2017.03.002. 28286317

[R84] Zhang R, Murray SB, Duval CJ, Wang DJJ, Jann K, 2024. Functional connectivity and complexity analyses of resting-state fMRI in pre-adolescents demonstrating the behavioral symptoms of ADHD. Psychiatry Res. 334, 115794. 10.1016/j.psychres.2024.115794. 38367454 PMC10947856

