# Supplementary material for: 7 tesla multiscale entropy analysis reveals increased resting-state complexity in key regions for fear and anxiety in spider-fearful individuals
Source: Neuroimage. Author manuscript; Available in PMC 2026 May 30. (PMC13221786; doi:10.1016/j.neuroimage.2025.121371)
Supplement: 1 [file NIHMS2170182-supplement-1.docx]

**Supplementary material S1: Exclusion of pan-regional low MSE outliers**

**
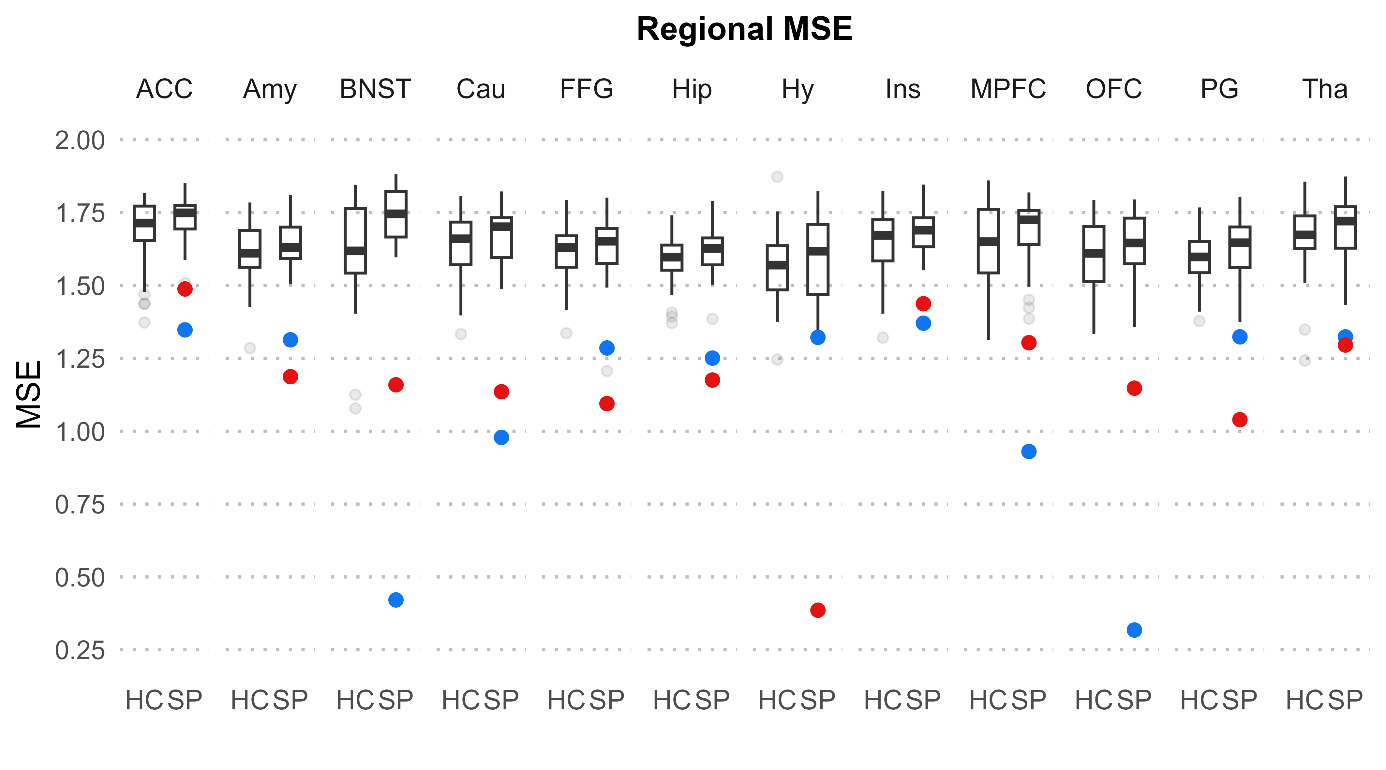
**

**Figure S1:** Boxplots of regional MSE averaged over all scales and stratified for group of the initial sample with N = 75. The datasets of the two subjects that were excluded from the analyses are coloured in red and blue. These were the only subjects that showed MSE outliers in all ROIs and values in BNST, Cau, Hy, MPFC, OFC, and PG, lower than the remaining 73 subjects across all ROIs. Note that the light grey dots indicate outliers of the boxplots other than those excluded, however, these outliers stem from various subjects, and never in more than 3 ROIs by a single subject.

**Supplementary material S2: MSE ANCOVA output**

**Table S1:** Mauchly's Test for Sphericity on average MSE

| **Effect** | ***W*** | ***p*** |
| --- | --- | --- |
| ROI | 0.001 | 1.03e-56 |
| Age:ROI | 0.001 | 1.03e-56 |
| Gender:ROI | 0.001 | 1.03e-56 |
| Group:ROI | 0.001 | 1.03e-56 |
| Scale | 5.9e-10 | 3.89e-263 |
| Age:Scale | 5.9e-10 | 3.89e-263 |
| Gender:Scale | 5.9e-10 | 3.89e-263 |
| Group:Scale | 5.9e-10 | 3.89e-263 |

**Table S2:** average MSE repeated-measures ANCOVA output (type III tests, Greenhouse Geisser corrected)

| **Effect** | ***F*** | ***df*** | ***p*** | ***η^2^*** |
| --- | --- | --- | --- | --- |
| Age | 0.932 | 1.00 | 0.338 | 0.013 |
| Gender | 1.368 | 1.00 | 0.246 | 0.019 |
| Group | 1.840 | 1.00 | 0.179 | 0.026 |
| ROI | 0.732 | 4.13 | 0.575 | 0.011 |
| Scale | 21.500 | 1.30 | **1.66e-06** | 0.238 |
| Age:ROI | 0.437 | 4.13 | 0.788 | 0.006 |
| Gender:ROI | 2.272 | 4.13 | 0.060 | 0.032 |
| Group:ROI | 1.707 | 4.13 | 0.146 | 0.024 |
| Age:Scale | 0.061 | 1.30 | 0.866 | 0.001 |
| Gender:Scale | 1.139 | 1.30 | 0.304 | 0.016 |
| Group:Scale | 0.178 | 1.30 | 0.739 | 0.003 |
| ROI:Scale | 7.858 | 99.00 | **4.22e-99** | 0.102 |
| Age:ROI:Scale | 0.906 | 99.00 | 0.737 | 0.013 |
| Gender:ROI:Scale | 1.495 | 99.00 | **0.001** | 0.021 |
| Group:ROI:Scale | 1.710 | 99.00 | **1.71e-05** | 0.024 |

**Supplementary material S3: group-wise Spearman correlations of the MSE of each ROI and scale with FSQ and STAItrait.**


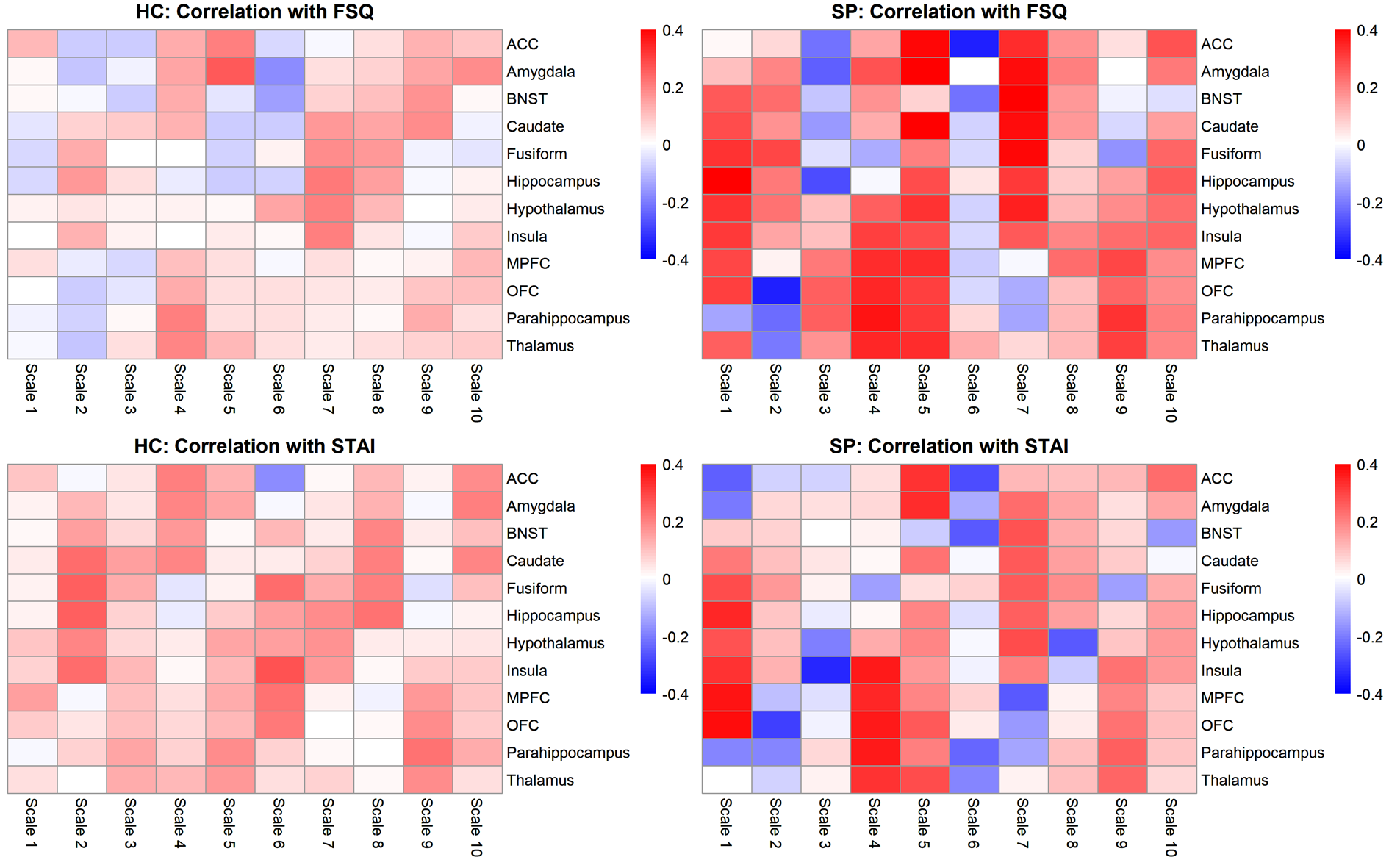


**Figure S2:** Correlations matrices of the regional MSE per scale. Upper panels depict correlations of MSE with FSQ, lower panels with STAItrait. Left-hand panels depict correlations of the HC group, right-hand panels of the SP group. The colour scale indicates Spearman rho coefficients; the ROIs are sorted alphabetically.

**Supplementary material S4: temporal signal-to-noise-ratio (tSNR)**

The methods are described in section 2.6 of the main article.

**Table S3:** Mean and standard deviation for the tSNR of each ROI

| **ROI** | ***tSNR (mean)*** | ***tSNR (sd)*** |
| --- | --- | --- |
| ACC | 75.87 | 12.03 |
| Amygdala | 55.17 | 10.20 |
| BNST | 55.59 | 16.05 |
| Caudate Nucleus | 69.22 | 13.83 |
| Fusiform Gyrus | 68.94 | 12.27 |
| Hippocampus | 63.15 | 8.61 |
| Hypothalamus | 33.63 | 14.03 |
| Insula | 72.46 | 10.61 |
| MPFC | 70.72 | 15.21 |
| OFC | 52.15 | 12.58 |
| Parahippocampal Gyrus | 50.83 | 9.41 |
| Thalamus | 60.50 | 10.42 |

**Supplementary material S5: Intrinsic connectivity**

The methods are described in section 2.6 of the main article.

**Table S4:** Mauchly's Test for Sphericity on average IC

| **Effect** | ***W*** | ***p*** |
| --- | --- | --- |
| ROI | 0.002 | 4.29e-51 |
| Age:ROI | 0.002 | 4.29e-51 |
| Sex:ROI | 0.002 | 4.29e-51 |
| Group:ROI | 0.002 | 4.29e-51 |

**Table S5:** average IC repeated-measures ANCOVA output (type III tests, Greenhouse Geisser corrected)

| **Effect** | ***F*** | ***df*** | ***p*** | ***η^2^*** |
| --- | --- | --- | --- | --- |
| Age | 3.183 | 1.00 | 0.079 | 0.044 |
| Sex | 6.767 | 1.00 | **0.011** | 0.089 |
| Group | 0.252 | 1.00 | 0.617 | 0.004 |
| ROI | 6.032 | 5.37 | **1.26e-05** | 0.080 |
| Age:ROI | 0.792 | 5.37 | 0.564 | 0.011 |
| Sex:ROI | 0.813 | 5.37 | 0.549 | 0.012 |
| Group:ROI | 0.675 | 5.37 | 0.654 | 0.010 |


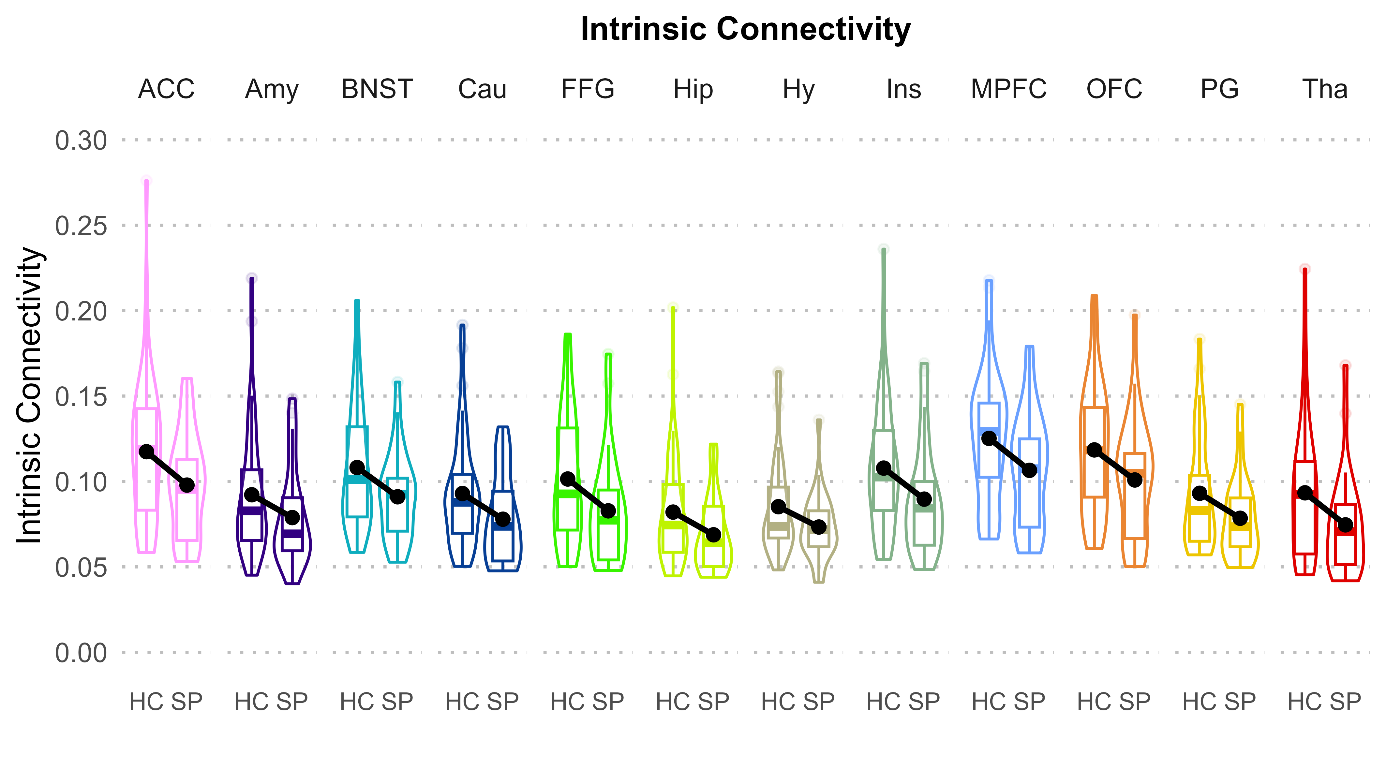


**Figure S3:** Averaged IC for each ROI stratified for group.
